# Cancer-Associated Fibroblasts Provide a Stromal Niche for Liver Cancer Organoids That Confers Trophic Effects and Therapy Resistance

**DOI:** 10.1016/j.jcmgh.2020.09.003

**Published:** 2020-09-12

**Authors:** Jiaye Liu, Pengfei Li, Ling Wang, Meng Li, Zhouhong Ge, Lisanne Noordam, Ruby Lieshout, Monique M.A. Verstegen, Buyun Ma, Junhong Su, Qin Yang, Ruyi Zhang, Guoying Zhou, Lucia Campos Carrascosa, Dave Sprengers, Jan N.M. IJzermans, Ron Smits, Jaap Kwekkeboom, Luc J.W. van der Laan, Maikel P. Peppelenbosch, Qiuwei Pan, Wanlu Cao

**Affiliations:** 1 Department of Gastroenterology and Hepatology, Erasmus MC-University Medical Center, Rotterdam, The Netherlands; 2 Department of Surgery, Erasmus Medical Center, University Medical Center, Rotterdam, The Netherlands; 3 Department of General Surgery, The Third People’s Hospital of Chengdu, Affiliated Hospital of Southwest Jiaotong University, Second Medical School of Chengdu, Chongqing Medical University, Chengdu, China

**Keywords:** Liver Tumor Organoids, Stromal Cells, Co-Culture, Cell–Cell Contact, Paracrine Effect, AFP, α-fetoprotein, α-SMA, α-smooth muscle actin, CAF, cancer-associated fibroblast, CCA, cholangiocarcinoma, CSC, cancer stem cell, DEN, N-nitrosodiethylamine, DMEM, Dulbecco’s modified Eagle medium, ECM, extracellular matrix, EGF, epidermal growth factor, EpCAM, epithelial cell adhesion molecule, FACS, fluorescence-activated cell sorter, FAP, fibroblast-associated protein, FCS, fetal calf serum, FGF, fibroblast growth factor, 5-FU, 5-fluorouracil, HCC, hepatocellular carcinoma, HGF, hepatocyte growth factor, IGF, insulin-like growth factor, IL, interleukin, NSG, NOD scid γ mouse, OBM, organoids basic medium, OEM, organoids expansion medium, PBS, phosphate-buffered saline, PCR, polymerase chain reaction, PDGFRA, platelet-derived growth factor receptor α, TCGA, The Cancer Genome Atlas, 3D, 3-dimensional, Wnt, wingless-related integration site

## Abstract

**Background & Aims:**

Cancer-associated fibroblasts (CAFs) play a key role in the cancer process, but the research progress is hampered by the paucity of preclinical models that are essential for mechanistic dissection of cancer cell–CAF interactions. Here, we aimed to establish 3-dimensional (3D) organotypic co-cultures of primary liver tumor–derived organoids with CAFs, and to understand their interactions and the response to treatment.

**Methods:**

Liver tumor organoids and CAFs were cultured from murine and human primary liver tumors. 3D co-culture models of tumor organoids with CAFs and Transwell culture systems were established in vitro. A xenograft model was used to investigate the cell–cell interactions in vivo. Gene expression analysis of CAF markers in our hepatocellular carcinoma cohort and an online liver cancer database indicated the clinical relevance of CAFs.

**Results:**

To functionally investigate the interactions of liver cancer cells with CAFs, we successfully established murine and human 3D co-culture models of liver tumor organoids with CAFs. CAFs promoted tumor organoid growth in co-culture with direct cell–cell contact and in a Transwell system via paracrine signaling. Vice versa, cancer cells secrete paracrine factors regulating CAF physiology. Co-transplantation of CAFs with liver tumor organoids of mouse or human origin promoted tumor growth in xenograft models. Moreover, tumor organoids conferred resistance to clinically used anticancer drugs including sorafenib, regorafenib, and 5-fluorouracil in the presence of CAFs, or the conditioned medium of CAFs.

**Conclusions:**

We successfully established murine and human 3D co-culture models and have shown robust effects of CAFs in liver cancer nurturing and treatment resistance.

## Graphical abstract

**Figure:**
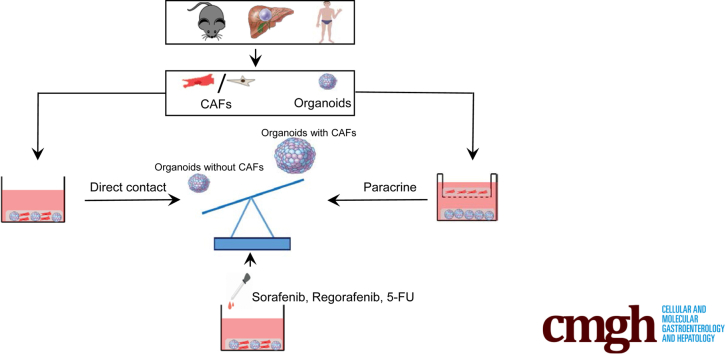

**Summary**

We have successfully established murine and human 3-dimensional co-culture models of primary liver tumor–derived organoids with cancer-associated fibroblasts. This model system enables the study of the interactions between tumor cells and the stromal compartment and the response to anticancer drugs.

Liver cancer is one of the most common and deadly malignancies worldwide, and currently there are limited treatment options available. Heterogeneity within and between liver tumors greatly complicates disease progression and treatment response.^1^ A subpopulation of cancer cells within tumors, termed cancer stem cells (CSCs), have been recognized to possess the capacity for both self-renewal and the potential for differentiation. This population of cells appears responsible for resistance to treatment in addition to tumor initiation and progression.^2^ Although tumor biology of liver cancer in general remains poorly understood, hopes for obtaining better understanding of this disease have been fostered by the recent development of 3-dimensional (3D) organoid culture technology. Such cultures, initially derived from tissue-resident stem/progenitor cells, embryonic stem cells, or induced pluripotent stem cells, has emerged as a new technology for stem cell research because they are capable of self-renewal and self-organization that recapitulates the functionality of the tissue-of-origin. Interestingly, this 3D culture system has been extended to culture a variety of primary cancer cells, providing insight into the role of CSCs in cancer progress.^3^ For liver cancer, tumor organoids that resemble hepatocellular carcinoma (HCC) or cholangiocarcinoma (CCA) have been cultured successfully from human tumor^4^ or mouse tumor models.^5^ In general, organoids are much easier to culture from CCA than HCC.

Cancer cells, in particular CSCs, actively interact with the tumor microenvironment. This microenvironment contains numerous cell types, including immune cells, fibroblasts, and endothelial cells, and various factors including signaling molecules and extracellular matrix (ECM).^6^ Among these components, a specialized group of fibroblasts called cancer-associated fibroblasts (CAFs) are considered to be of unusual importance to tumor development. Previous studies have identified several CAF markers including α-smooth muscle actin (α-SMA), fibroblast-associated protein (FAP), vimentin, fibroblast-specific protein 1 (FSP1), CD29, caveonin 1 (CAV1), desmin, platelet-derived growth factor receptor α (PDGFRA), platelet-derived growth factor receptor β (PDGFRB), gremlin 1, collagen type I α 1, periostin (COL1A1), and C-X-C motif chemokine 12 (CXCL12).^7^, ^8^, ^9^, ^10^, ^11^, ^12^, ^13^, ^14^ CAFs can support tumor growth, metastasis, and the formation of cancer stem cell niches, and mediate immunosuppression and drug resistance by directly interacting with cancer cells or secreting a panel of factors and nutrients.^15^ More than 80% of HCC patients have a background of cirrhosis,^16^ and these livers are enriched with activated fibroblasts as a result of the chronic inflammation that characterizes this disease. Thus, CAFs are assumed to play a prominent role in liver cancer even in the absence of formal proof.

In this study, we first developed a 3D co-culture system of primary liver tumor–derived organoids with CAFs of mouse or human origin. By using this system, we investigated the reciprocal interactions of cancer cells and CAFs, and the role that the CAF niche provided with respect to the nurturing of cancer cells and their importance for treatment resistance of liver cancer cells.

## Results

### Evidence for the Potential Clinical Significance of CAFs in Liver Cancer

**Figure 1:**
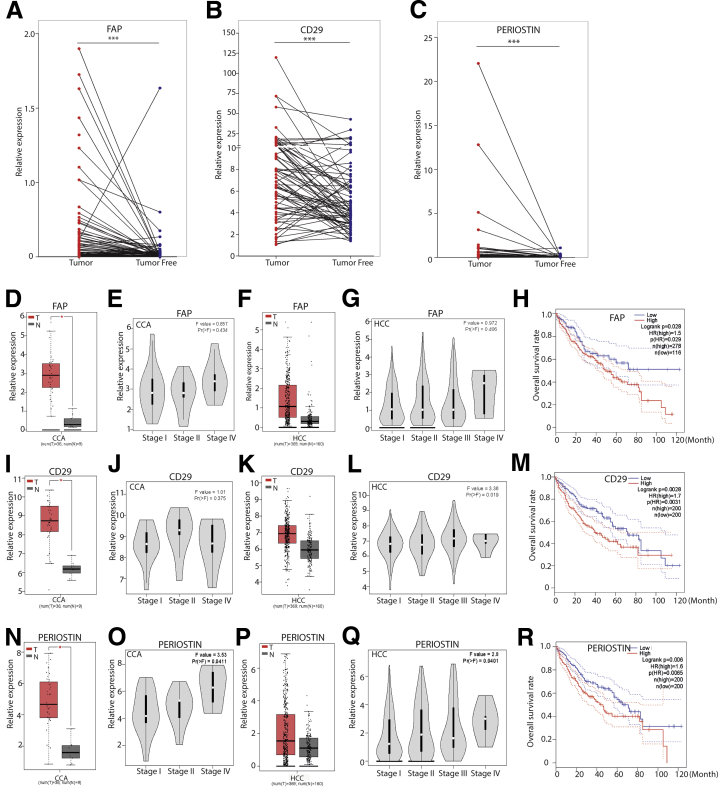
**Bioinformatics analysis between FAP, CD29, and periostin gene expression and clinical relevance in liver cancer.** (*A–C*) Gene expression of CAF markers FAP, CD29, and periostin in tumors compared with paired adjacent tumor-free liver tissues in our HCC cohort (N = 75 HCC, Mann–Whitney *U* tests). ∗∗∗*P* < .001. (*D*, *I*, and *N*) Gene expression of CAF markers FAP, CD29, and periostin in CCA compared with normal liver tissues in an online TCGA database (n = 9 for normal liver tissue, n = 36 for tumor tissue; 1-way analysis of variance). ∗*P* < .05. (*F*, *K*, and *P*) Gene expression of CAF markers FAP, CD29, and periostin in HCC compared with normal liver tissues in an online TCGA database (n = 160 for normal liver tissue, n = 369 for tumor tissue; 1-way analysis of variance). ∗*P* < .05. (*E*, *J*, and *O*) The expression of FAP, CD29, and periostin in different tumor stages of CCA (n = 36, 1-way analysis of variance). (*G*, *L*, and *Q*) The expression of FAP, CD29, and periostin in different tumor stages of HCC (n = 369, 1-way analysis of variance). (*H*, *M*, and *R*) Overall survival assessed using the online TCGA database at www.gepia.com. The differences in survival related to CAF markers CD29, FAP, and periostin messenger RNA expression were compared in each group involving all patients (Log-rank test, FAP [n = 36 for CCA, n = 358 for HCC]; CD29 [n = 36 for CCA, n = 364 for HCC]; periostin [n = 36 for CCA, n = 364 for HCC]). *Dotted line* indicates the 95% CI. HR, hazard ratio; N, normal liver tissue; T, tumor tissue.

**Figure 2:**
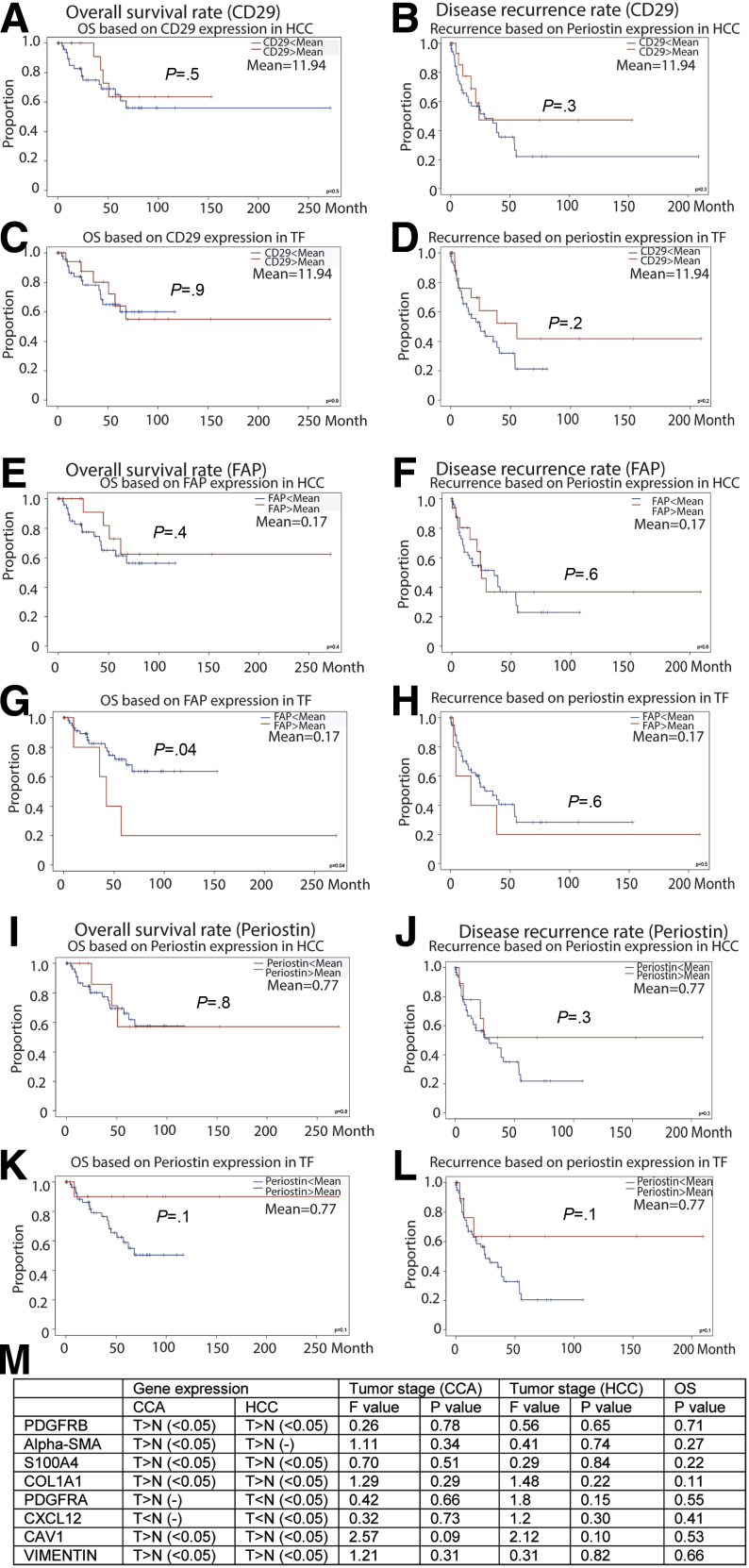
**Survival and recurrence analysis based on the gene expression of CD29, FAP, and periostin in our HCC cohort and bioinformatics analysis of other CAF markers in the GEPIA online database.** (*A–L*) Overall survival and disease-free rate based on the gene expression of CD29, FAP, and periostin in tumor tissue or tumor-free liver tissue of our HCC patients (Kaplan–Meier analysis, N = 75). (*M*) Bioinformatics analysis of PDGFRB, α-SMA, S100A4, COL1A1, PDGFRA, CXCL12, CAV1, and vimentin in the GEPIA database. The gene expression of these markers in tumor and normal liver tissue (CCA: n = 9 for normal liver tissue; n = 36 for tumor tissue; HCC: n = 160 for normal liver tissue; n = 369 for tumor tissue) was assessed by 1-way analysis of variance. Gene expression of these markers in different stages of liver tumors (CCA: n = 36; HCC: n = 369) was assessed by 1-way analysis of variance. The differences in survival related to CAF markers PDGFRB, α-SMA, FSP1, COL1A1, PDGFRA, CXCL12, CAV1, and vimentin messenger RNA expression were compared in each group involving all patients (Log-rank test, n = 36 for CCA, n = 364 for HCC). (-) Without a statistically significant difference. CAV1, caveolin 1; COL1A1, collagen type I α 1; CXCL12, C-X-C motif chemokine ligand 12; FSP1, fibroblast-specific protein 1; N, normal liver tissue; OS, overall survival; PDGFRB, platelet-derived growth factor receptor β; T, tumor tissue; TF, tumor free.

We first examined the potential clinical relevance of CAFs in liver cancer patients. We quantified the messenger RNA expression of 3 well-recognized CAF markers including FAP,^17^^,^^18^ CD29,^19^^,^^20^ and periostin^21^^,^^22^ in our HCC patient cohort. Their expression was increased significantly in tumors compared with adjacent liver tissues of the same patients (N = 75) (Figure 1*A–C*). We next analyzed the expression of these CAF markers using The Cancer Genome Atlas (TCGA) online database. Consistently, FAP, CD29, and periostin are up-regulated in tumor compared with normal liver tissues (n = 196 for normal liver tissue; n = 405 for tumor tissue, including 369 HCC and 36 CCA) (Figure 1*D*, *F*, *I*, *K*, *N*, and *P*). This up-regulation is more apparent in late stages of liver cancer (Figure 1*E*, *G*, *J*, *L*, *O*, and *Q*). Importantly, high expression of FAP, CD29, or periostin in tumor tissues is associated significantly with poor overall survival of the patients (Figure 1*H*, *M*, and *R*). The number of patients in our HCC cohort was too small for a powerful statistical analysis of CAF markers in association with cancer-specific survival. Some of these markers showed similar trends in relation to patient survival in our cohort as observed in the TCGA data set, although they are not fully in accordance with the results from the TCGA database (Figure 2*A–L*). Analysis of additional CAF markers showed the up-regulation of several other markers in tumor, although their expression was not associated with patient survival (Figure 2*M*). These results provided some evidence for the potential clinical relevance of CAFs in liver cancer and prompted us to establish experimental models for further investigation.

### Construction of 3D Co-culture Systems of Liver Tumor Organoids With CAFs

**Figure 3:**
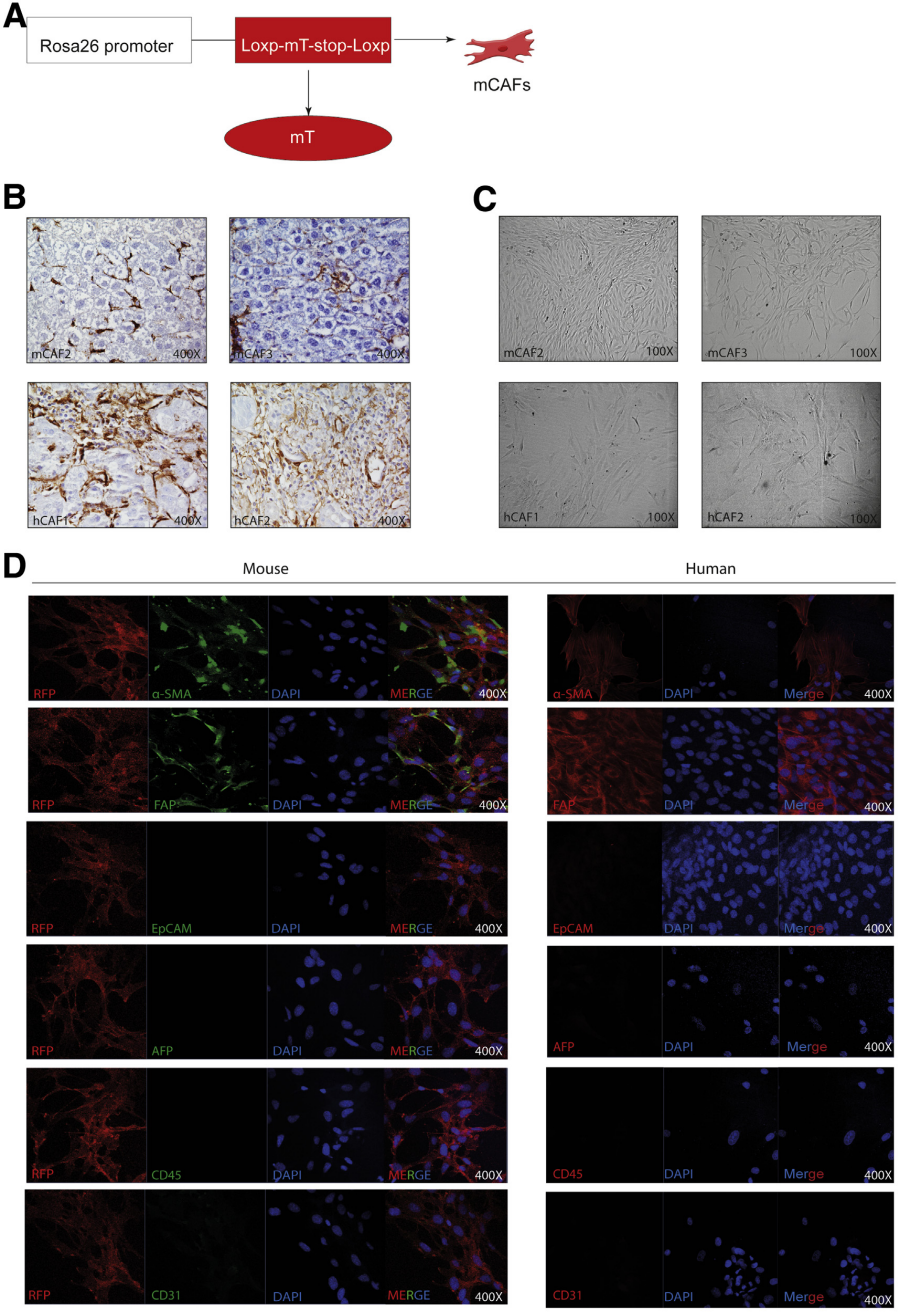
**Establishment of CAFs.** (*A*) Rosa26-mT mouse treated with DEN for 17 weeks, and waiting 30 weeks for tumor formation. Then mouse CAFs were cultured according to our protocol. (*B*) Representative immunohistochemistry staining of α-SMA in mouse and human primary tissue (magnification, 400×). (*C*) Representative image of established human and mouse CAFs (magnification, 100×). (*D*) Representative immunofluorescence staining of α-SMA, FAP, EpCAM, AFP, CD45, and CD31 in mouse and human CAFs (magnification, 400×). DAPI, 4′,6-diamidino-2-phenylindole; hCAF, human cancer associated fibroblast; mCAF, mouse cancer associated fibroblast; mT, membrane tomato; RFP, red fluorescent protein.

For studying the interaction between cancer cells and CAFs, we first explored the construction of 3D organotypic co-culture systems of liver tumor organoids with CAFs. We established 6 mouse tumor organoids from carcinogen N-nitrosodiethylamine (DEN)-induced mouse liver tumors and 4 human CCA tumor organoids from resected patient CCA tumors as previously described.^4^^,^^5^ CAFs were isolated and cultured from DEN-induced liver tumors of red fluorescence-expressing Rosa 26-membrane tomato mice (Figure 3*A*), and tumors of HCC and CCA patients (Figure 3*B*). As a result, 2 mouse CAFs (2 of 6 mice), 6 human CAFs (2 of 3 CCA and 4 of 10 HCC) were established. CAFs were enriched by plastic adherence and propagated in culture. Both mouse and human CAFs show an elongated, spindle-like morphology (Figure 3*C*). Immunofluorescence staining confirmed that most CAFs were positive for α-SMA and FAP (Figure 3*D*). We excluded the presence of other cell types including cancer cells, immune cells, and endothelial cells by staining with the corresponding markers α-fetoprotein (AFP), epithelial cell adhesion molecule (EpCAM), cluster of differentiation 45 (CD45), and CD31 (Figure 3*D*).

**Figure 4:**
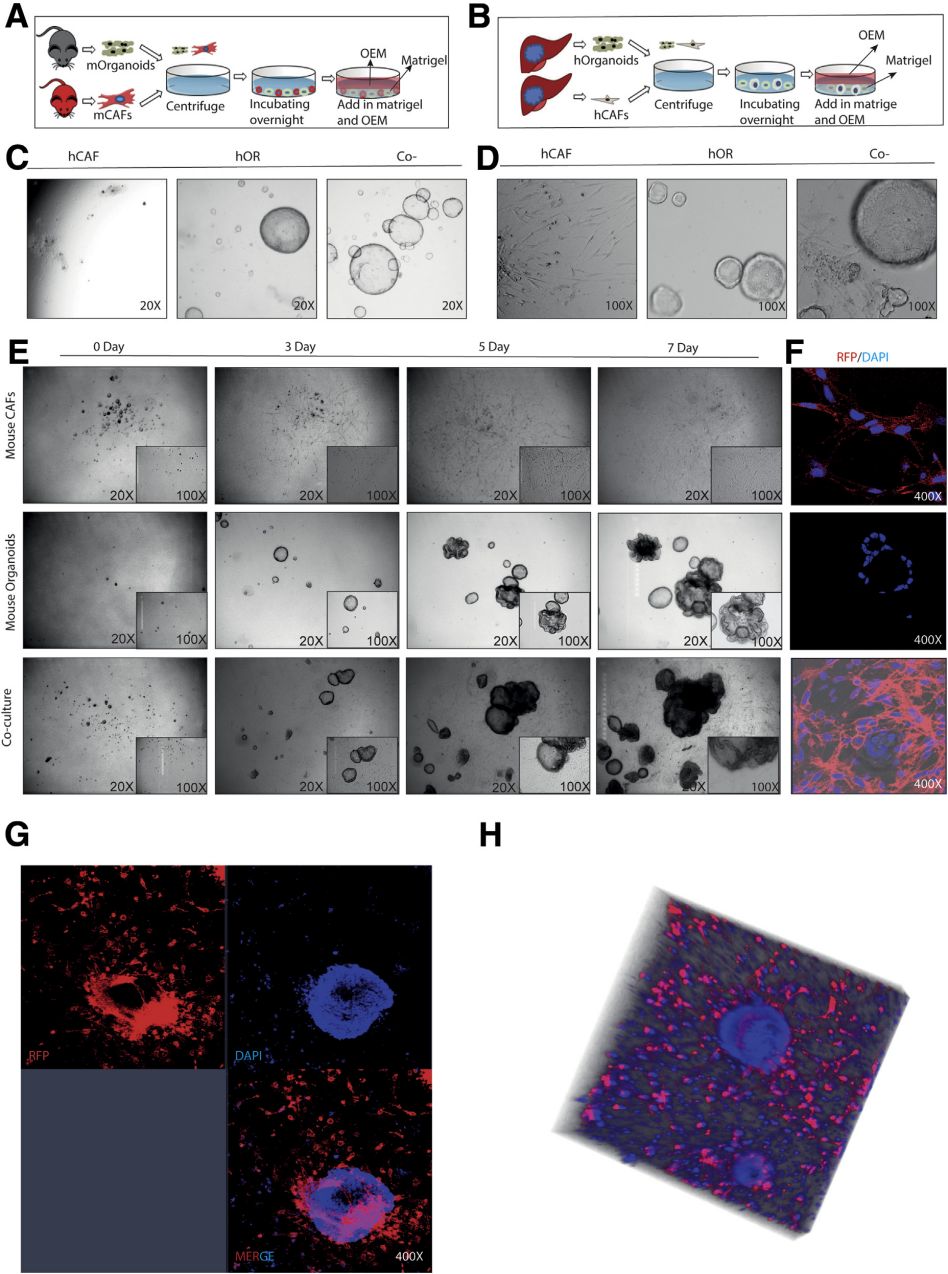
**Establishment of organoid and CAF co-culture models of mouse and human origins.** (*A* and *B*) Schematic illustration of the co-culture models of murine and human origins. (*C* and *D*) Representative image of human CAFs, human organoids, and co-cultures at day 10 (*C*, magnification, 20×; *D*, magnification, 100×). (*E*) Representative image of mouse CAFs, mouse organoids, and co-cultures from day 0 to day 7 (magnification, 20×; *inset*: magnification, 100×). (*F*) Representative immunofluorescence staining of mouse CAFs, mouse organoids, and co-cultures (magnification, 400×). (*G*) Representative confocal image of mouse organoids and CAF co-culture model (magnification, 400×). (*H*) Representative 3D reconstruction of Z-stack of mouse organoids and CAF co-culture model. DAPI, 4′,6-diamidino-2-phenylindole; hCAF, human cancer associated fibroblast; hOR, human organoid; mCAF, mouse cancer associated fibroblast; mOrganoids, mouse organoids; RFP, red fluorescent protein.

We successfully established the murine and human 3D co-cultures of tumor organoids and CAFs (Figure 4*A–E*). However, the co-cultured organoids and CAFs were not derived from the same mice or patients. After 3 days in co-culture, CAFs became further elongated and gradually formed a net-like structure that encircled organoids (Figure 4*E*). Corresponding immunofluorescence images of the culture system of mouse origin are shown because these CAFs were derived from red fluorescent protein expression in murine liver tumor (Figure 4*F*). By using immunofluorescence staining and 3D reconstruction of the Z-stack of confocal images, we further confirmed that CAFs surrounded the organoids closely (Figure 4*G* and *H*).

### CAFs Promote the Growth of Organoids in Co-culture

**Figure 5:**
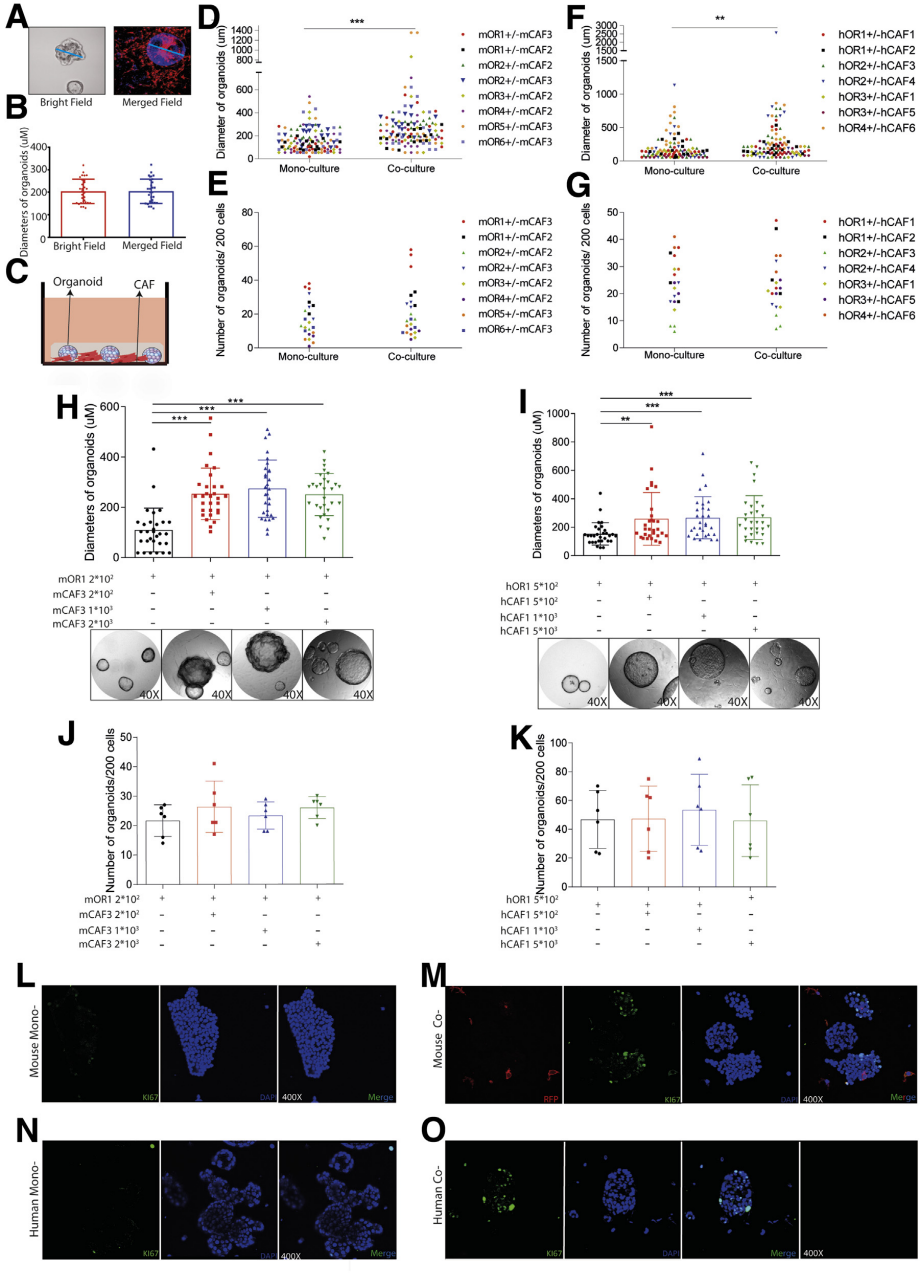
**The effects of CAFs on tumor organoid formation and growth.** (*A* and *B*) Measuring the diameter of organoids under immunofluorescence and bright field vision (n = 6, 5 organoids for each well randomly were measured). (*C*) Mouse or human tumor organoids cultured with or without corresponding CAFs. (*D*) Diameters of mouse organoids cultured with or without mouse CAFs (n = 8 experimental settings with 3 biological replicates for each; 5 organoids for each well randomly were measured). (*E*) Number of mouse organoids cultured with or without mouse CAFs (n = 8 experimental settings with 3 biological replicates for each). (*F*) Diameters of human organoids cultured with or without human CAFs (n = 7 experimental settings with 3 biological replicates for each; 5 organoids for each well randomly were measured). (*G*) Number of human organoids cultured with or without mouse CAFs (n = 7 experimental settings with 3 biological replicates for each). (*H* and *I*) Diameters of formed organoids in mono- or co-cultures with different concentrations between organoids and CAFs (n = 6; 5 organoids for each well randomly were measured). (*J* and *K*) The number of formed organoids in mono- or co-cultures with different concentrations between organoids and CAFs (n = 6). (*L*) Ki67 staining for mouse organoid mono-culture (magnification, 400×). (*M*) Ki67 staining for mouse organoids and CAF co-culture (magnification, 400×). (*N*) Ki67 staining for human organoid mono-culture (magnification, 400×). (*O*) Ki67 staining for human organoids and CAF co-culture (magnification, 400×). (*B* and *D–K*) Data are expressed as means ± SD. Mann–Whitney *U* tests. ∗∗*P* < .01, ∗∗*P* < .05, ∗∗∗*P* < .001. hCAF, human cancer associated fibroblast; hOR, human organoid; mCAF, mouse cancer associated fibroblast; mOR, mouse organoid; RFP, red fluorescent protein.

After co-culturing digested single murine organoid cells with CAFs for 7 days and those of human origin for 14 days, we counted the number of formed organoids and randomly measured the diameter of 5 organoids in each well (Figure 5*A*). We verified the accuracy of our measurement by measuring the diameter both under immunofluorescence and bright field vision (Figure 5*B*). We found that co-culturing CAFs enlarged the size of formed organoids, but did not affect the number (Figure 5*C–G*). This effect already was apparent at a 1:1 ratio input of organoid and CAF cells, but was not enhanced by further increasing the input of CAFs (Figure 5*H–K*). Enhanced expression of the cell proliferation marker Ki67 in co-cultured organoids further supports this promoting effect (Figure 5*L–O*). Therefore, these results suggest that CAFs may not regulate the efficiency of organoid initiation, but promote the growth of formed organoids in the co-culture system.

### Reciprocal Enhancement of CAFs and Tumor Organoid Growth Through Paracrine Signaling

**Figure 6:**
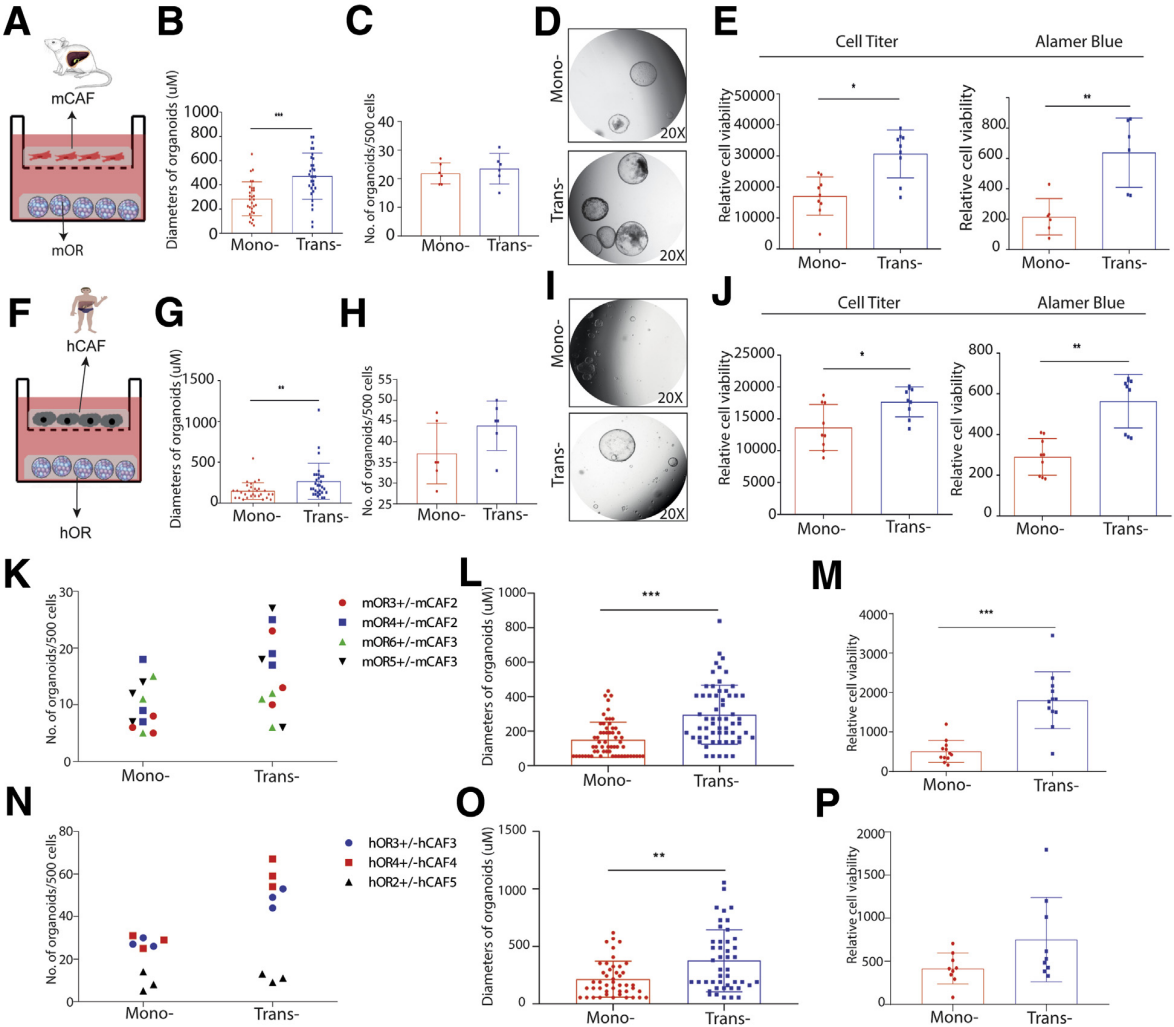
**The effects of CAFs on organoids on a Transwell platform.** (*A*) Schematic illustration of a Transwell culture platform for mouse cells. (*B*) Diameters of mouse organoids on a Transwell platform with or without CAFs (n = 6; 5 organoids for each well randomly were measured). (*C*) Number of mouse organoids on a Transwell platform with or without CAFs (n = 6). (*D*) Representative images of mono-cultured, co-cultured mouse organoids. (*E*) Growth of mouse liver tumor organoids determined by CellTiter (n = 9) and Alamar Blue Assay (n = 6). (*F*) Schematic illustration of a Transwell culture platform for human cells. (*G*) Diameters of human organoids on a Transwell platform with or without CAFs (n = 6; 5 organoids for each well randomly were measured). (*H*) Number of human organoids on a Transwell platform with or without CAFs (n = 6). (*I*) Representative images of mono-cultured, co-cultured human organoids on a Transwell platform. (*J*) Growth of human organoids determined by CellTiter and Alamar Blue Assay (n = 9). (*K*) The number of formed mouse tumor organoids in the presence or absence of CAFs in a Transwell system (n = 4 experimental settings with 3 biological replicates for each, Mann–Whitney *U* tests). (*L*) The size of formed mouse tumor organoids in the presence or absence of CAFs in a Transwell system (n = 4 experimental settings with 3 biological replicates for each; 5 organoids for each well randomly were measured). (*M*) Growth of mouse liver tumor organoids determined by Alamar Blue Assay (n = 4 experimental settings with 3 biological replicates for each). (*N*) The number of formed human tumor organoids in the presence or absence of CAFs in a Transwell system (n = 3 experimental settings with 3 biological replicates for each, Mann–Whitney *U* tests). (*O*) The size of formed human tumor organoids in the presence or absence of CAFs in a Transwell system (n = 3 experimental settings with 3 biological replicates for each; 5 organoids for each well were measured randomly). (*P*) Growth of mouse liver tumor organoids determined by Alamar Blue Assay (n = 3 experimental settings with 3 biological replicates for each). (*B*, *C*, *E*, *G*, *H*, *J*, *L*, *M*, *O*, and *P*) Data are expressed as means ± SD. Mann–Whitney *U* tests. ∗*P* < .05, ∗∗*P* < .01, ∗∗∗*P* < .001. hCAF, human cancer associated fibroblast; hOR, human organoid; mCAF, mouse cancer associated fibroblast; mOR, mouse organoid.

**Figure 7:**
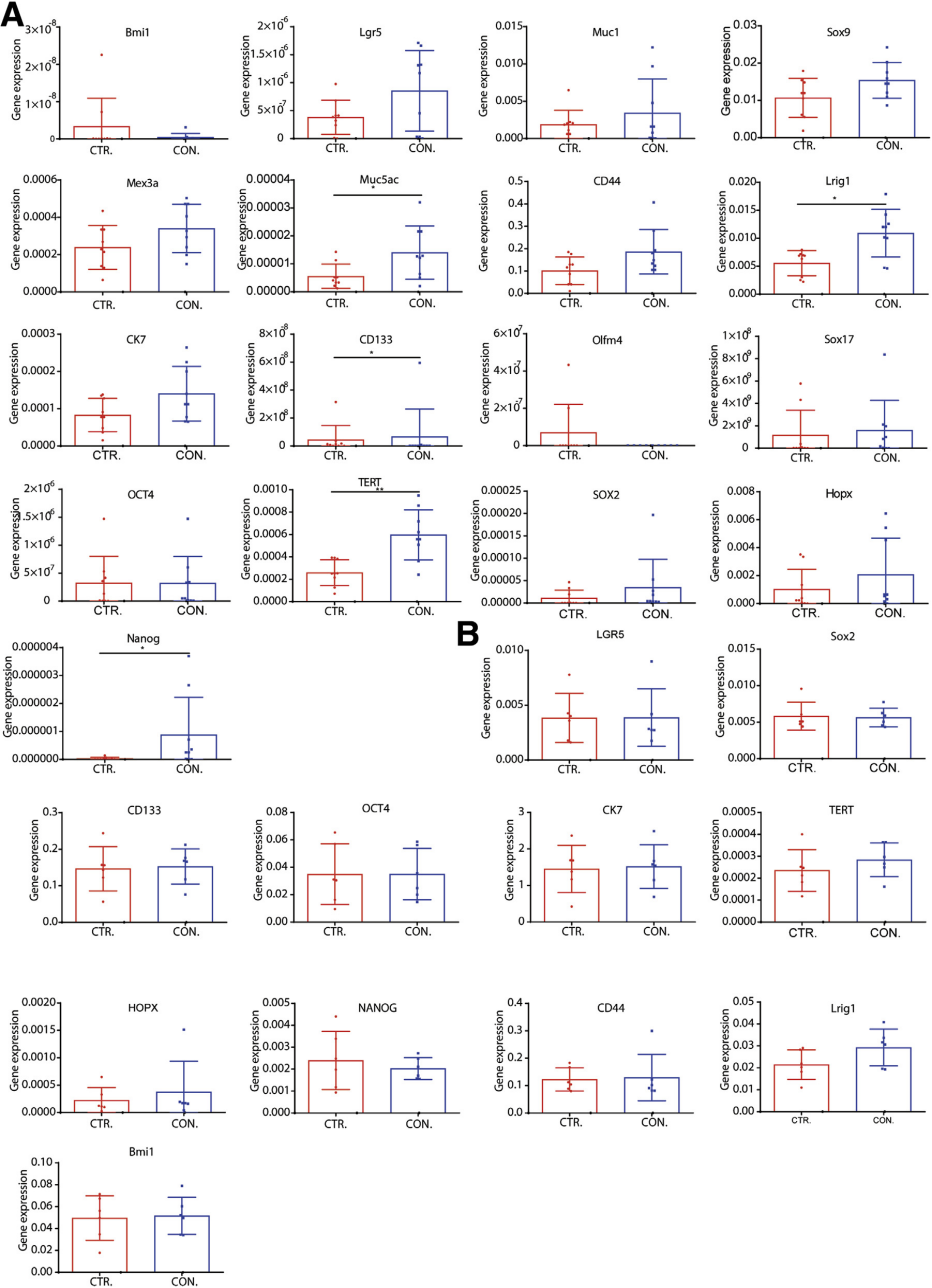
**The expression profile of stem cell markers in tumor organoids.** (*A*) Stem cell marker expression of mouse organoids in the presence or absence of CAF-conditioned medium (n = 9). (*B*) Stem cell marker expression of human organoids in the presence or absence of CAF-conditioned medium (n = 6). (*A* and *B*) Data are expressed as means ± SD. Mann–Whitney *U* tests. ∗*P* < .05, ∗∗*P* < .01. CON, conditioned; CTR, control.

The aforementioned results were shown in the co-culture system, but this does not exclude the possibility of paracrine effects. To investigate this, we established a Transwell system in which CAFs were seeded on the top and organoids on the bottom layer (Figure 6*A*). After incubation for 10 days, we found that CAFs did not affect the number, however, reminiscent to co-culture, increased the diameter of formed organoids in the setting of cells of mouse origin (Figure 6*B–D*). Cell Titer Assay and Alamar Blue Assay further confirmed these results (Figure 6*E*). The same results were observed in the setting of other combinations of mouse cells as well as cells of human origin (Figure 6*F–P*). Interestingly, several stem cell markers including Lrig1, Muc5ac, CD133, TERT, and NANOG were up-regulated in mouse organoids by the paracrine effect of CAFs (Figure 7*A*). However, this was not observed in human organoids (Figure 7*B*).

**Figure 8:**
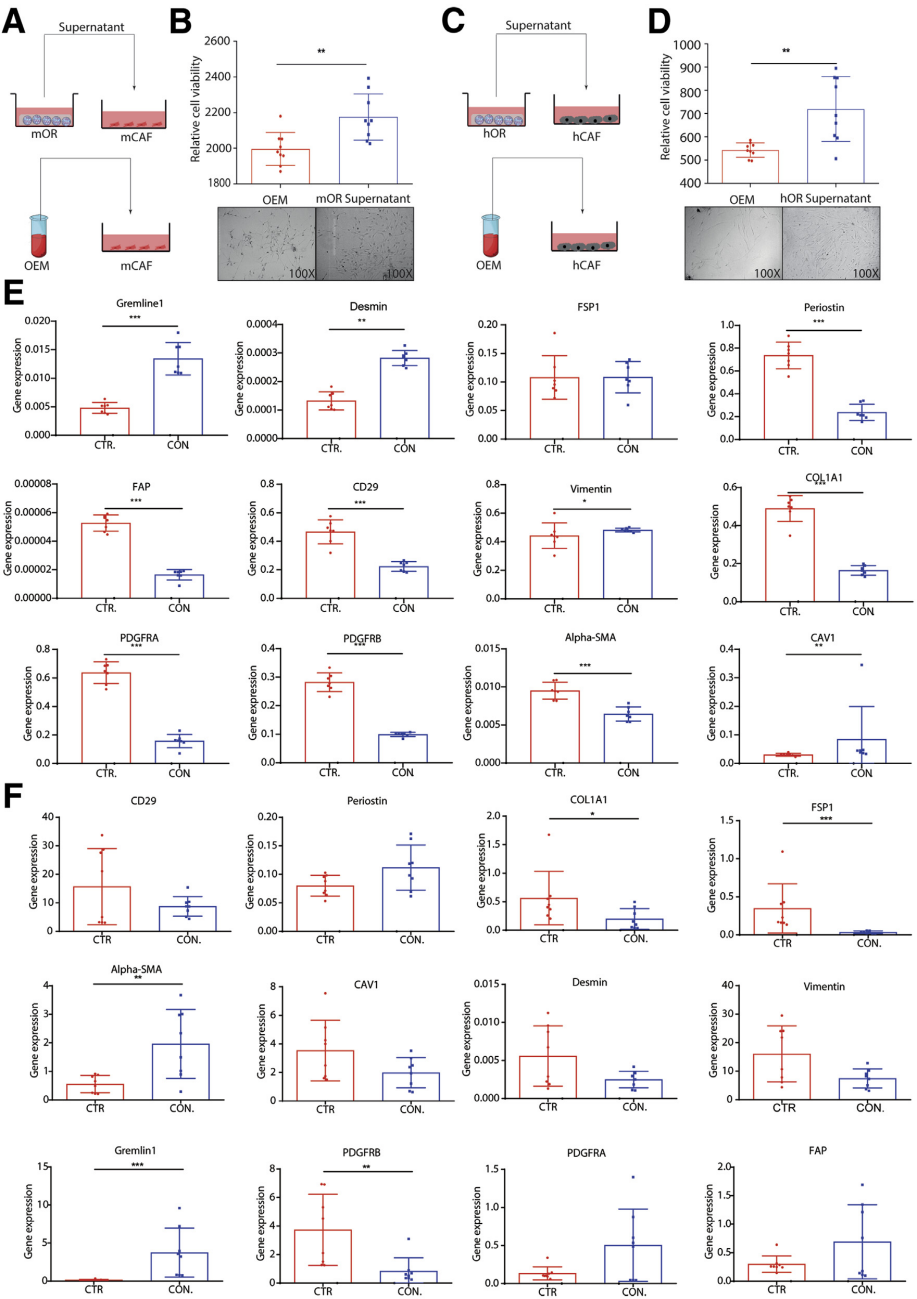
**Supernatant of organoids on the growth, morphology, and gene expression of CAFs.** (*A* and *B*) Growth of mouse CAFs in the presence or absence of organoid conditioned medium (n = 9). (*C* and *D*) Growth of human CAFs in the presence or absence of organoid conditioned medium (n = 9). (*E*) Expression profile of mouse CAFs markers in the presence or absence of organoid conditioned medium (n = 8). (*F*) Expression profile of human CAF markers in the presence or absence of organoid conditioned medium (n = 8). (*B* and *D*–*F*) Data are presented as means ± SD. Mann–Whitney *U* tests. ∗*P* < .05, ∗∗*P* < .01, ∗∗∗*P* < .001. CTR, control; CON, conditioned; FSP1, fibroblast-specific protein 1; hCAF, human cancer associated fibroblast; hOR, human organoid; mCAF, mouse cancer associated fibroblast; mOR, mouse organoid.

Next, we examined the reverse effect by exposing CAFs to the conditioned medium of tumor organoids (Figure 8*A* and *C*). We found that soluble factors from tumor organoids significantly promoted the growth of CAFs (Figure 8*B* and *D*). Profiling a panel of potential CAFs markers showed that gremlin 1 was up-regulated in both mouse and human CAFs (Figure 8*E* and *F*). Previous studies have documented that gremlin 1 suppresses the function of bone morphogenetic proteins that may support cancer stemness.^23^ Thus, CAFs and organoids reciprocally facilitate their growth at least partially through paracrine signaling.

### CAFs Promote the Growth of Organoid-Formed Tumors in Mice

**Figure 9:**
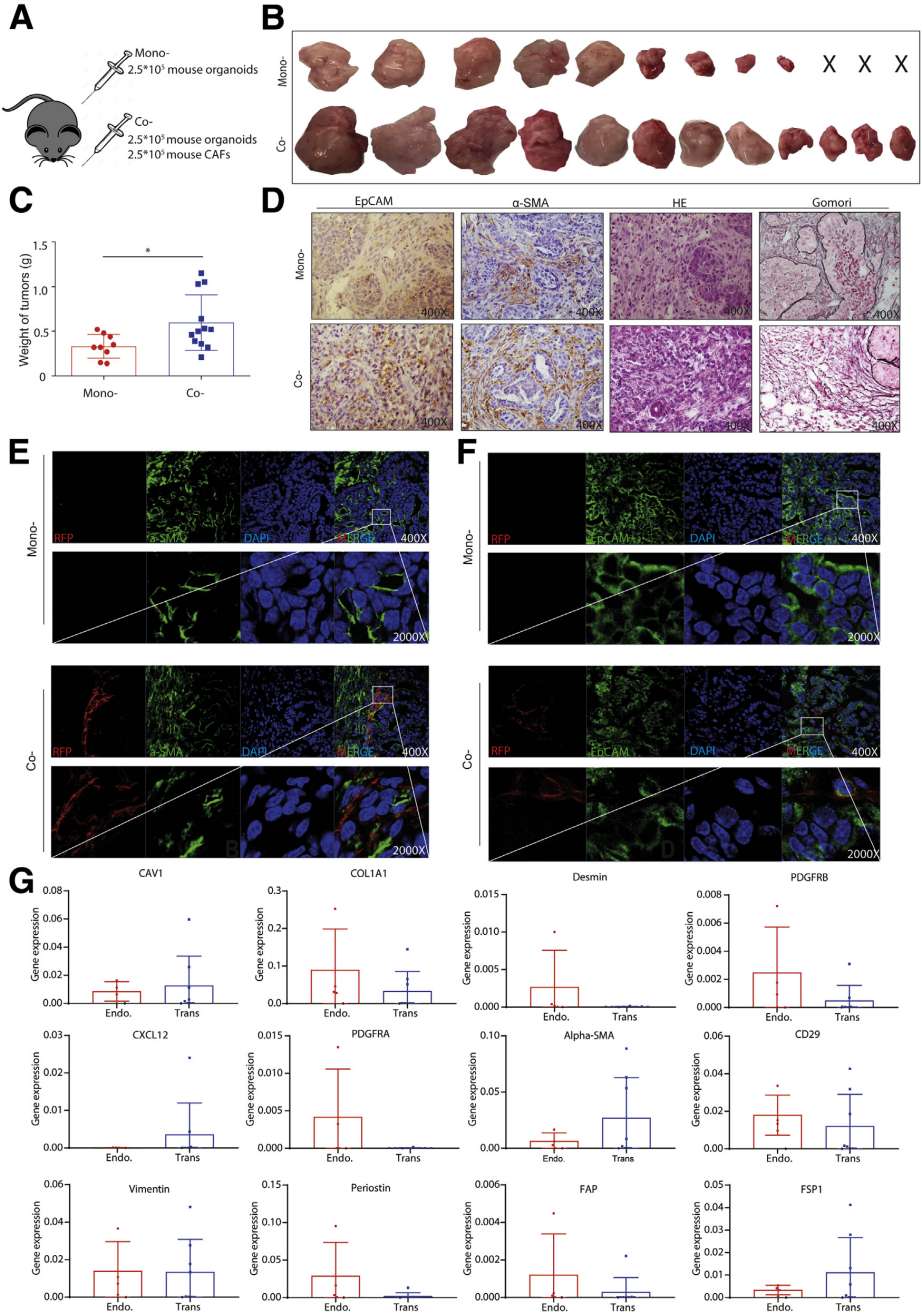
**Mouse CAFs promote the growth of mouse organoid-formed tumors in vivo.** (*A*) Mouse tumor organoids (2.5 × 10^5^) together with or without 2.5 × 10^5^ mouse CAFs were transplanted into NSG mice. (*B*) Representative pictures show the tumors from mono- and co-transplantation. (*C*) The weight of tumors from mono- or co-transplantation (n = 9 for xenografts from organoid transplantation only, n = 12 for xenografts from CAFs and organoid co-transplantation; ∗*P* < .05). (*D*) The representative immunohistochemistry staining of EpCAM, α-SMA, H&E, and Gomori for tumors from mono- or co-transplantation (magnification, 400×). (*E*) The representative confocal image of α-SMA expression for tumors from mono- or co-transplantation (magnification, 400×; *inset*: magnification, 2000×). (*F*) The representative confocal image of EpCAM expression for tumors from mono- or co-transplantation (magnification, 400×; *inset*: magnification, 2000×). (*G*) Expression profile of CAF markers for transplanted and endogenously recruited mouse CAFs (endogenous, n = 4; transplanted, n = 8). (*C* and *G*) Data are presented as means ± SD. Mann–Whitney *U* tests. α-SMA, alpha smooth actin; CAFs, cancer associated fibroblasts; DAPI, 4′,6-diamidino-2-phenylindole; Endo, endogenous; EpCAM, epithelial cell adhesion molecule; H&E, hematoxylin and eosin; NSG, NOD scid gamma mouse; RFP, red fluorescent protein; Trans, transplant.

We previously have shown that liver tumor organoids are capable of forming tumors on subcutaneous transplantation in immunodeficient mice.^5^ We thus investigated the effects of CAFs on organoid-based tumor formation and growth in vivo (Figure 9*A*). We found that co-transplantation of organoids with CAFs lead to more efficient tumor formation (12 of 12) than transplanting mouse organoids alone (9 of 12) (Figure 9*B*). More importantly, co-transplantation resulted in much larger tumors compared with transplanting organoids alone (tumor weight, 0.60 ± 0.31 g [n = 12] vs 0.33 ± 0.13 g [n = 9]; *P* < .05) (Figure 9*C*). Immunohistochemistry and immunofluorescence staining confirmed the presence of CAFs in the tumor tissue of mice co-transplanted with CAFs (Figure 9*D–F*). Interestingly, CAFs also were present abundantly in the tumors of control mice transplanted with organoids alone (Figure 9*D* and *E*), suggesting that tumor organoids and the formed tumors can recruit endogenous CAFs efficiently. Because the transplanted CAFs express red fluorescent protein, we were able to separate the transplanted CAFs and endogenous CAFs using fluorescence-activated cell sorter (FACS). The expression levels of some CAF markers indeed are substantially different, but the pattern is not very clear (Figure 9*G*).

**Figure 10:**
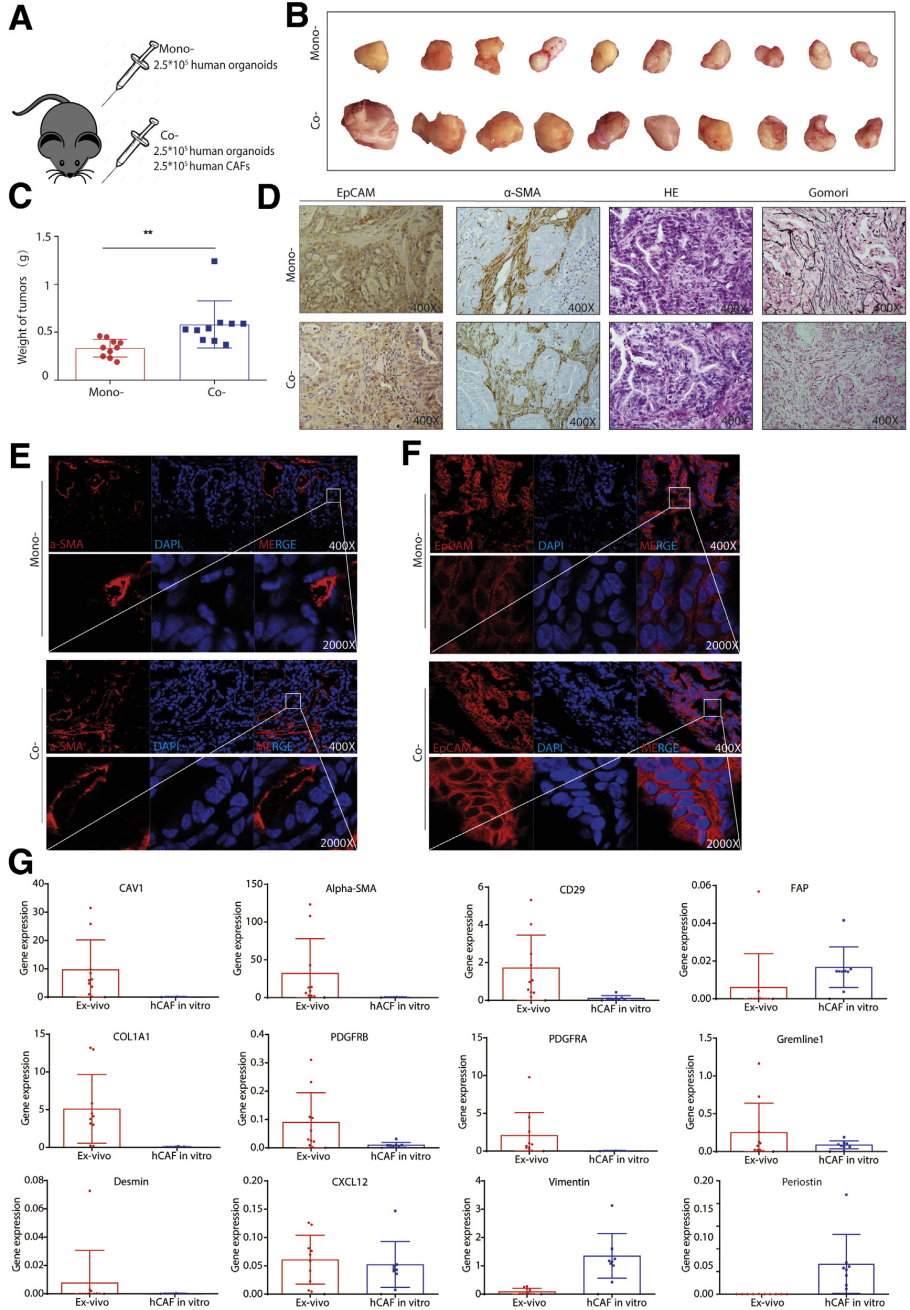
**Human CAFs promote the growth of patient CCA organoid-formed tumors in vivo.** (*A*) Human tumor organoids (2.5 × 10^5^) together with or without 2.5 × 10^5^ human CAFs were transplanted into NSG mice. (*B*) Representative pictures show the tumors from mono- and co-transplantation. (*C*) The weight of tumors from mono- or co-transplantation (n = 10 for both groups; ∗∗*P* < .01). (*D*) The representative immunohistochemistry staining of EpCAM, α-SMA, H&E, and Gomori for tumors from mono- or co-transplantation (magnification, 400×). (*E*) The representative confocal image of α-SMA expression for tumors of mono- or co-transplantation (magnification, 400×; *inset*: magnification, 2000×). (*F*) The representative confocal image of EpCAM expression for tumors from mono- or co-transplantation (magnification, 400×; *inset*: magnification, 2000×). (*G*) Expression profile of CAF markers for in vivo educated human CAFs from xenograft tumors compared with in vitro cultured CAFs (educated, n = 10; in vitro, n = 8). (*C* and *G*) Data are presented as means ± SD. Mann–Whitney *U* tests. α-SMA, alpha smooth actin; H&E, hematoxylin and eosin; DAPI, 4′,6-diamidino-2-phenylindole; EpCAM, epithelial cell adhesion molecule; hCAF, human cancer associated fibroblast; NSG, NOD scid gamma mouse.

Consistently, co-transplantation with human CAFs also promoted tumor formation and growth of patient CCA organoids in mice (Figure 10*A–C*). Immunohistochemistry and immunofluorescence staining confirmed the presence of CAFs in the tumors (Figure 10*D* and *F*). We next isolated the in vivo educated human CAFs from the tumors and compared their gene expression with in vitro cultured CAFs. We found a distinct expression pattern of the CAF markers, showing a trend of enhanced expression of CAF markers in tumor educated CAFs (Figure 10*G*). Taken together, CAFs support organoid-based tumor formation and growth in vivo.

### CAFs Protect Tumor Organoids From Drug Treatment

**Figure 11:**
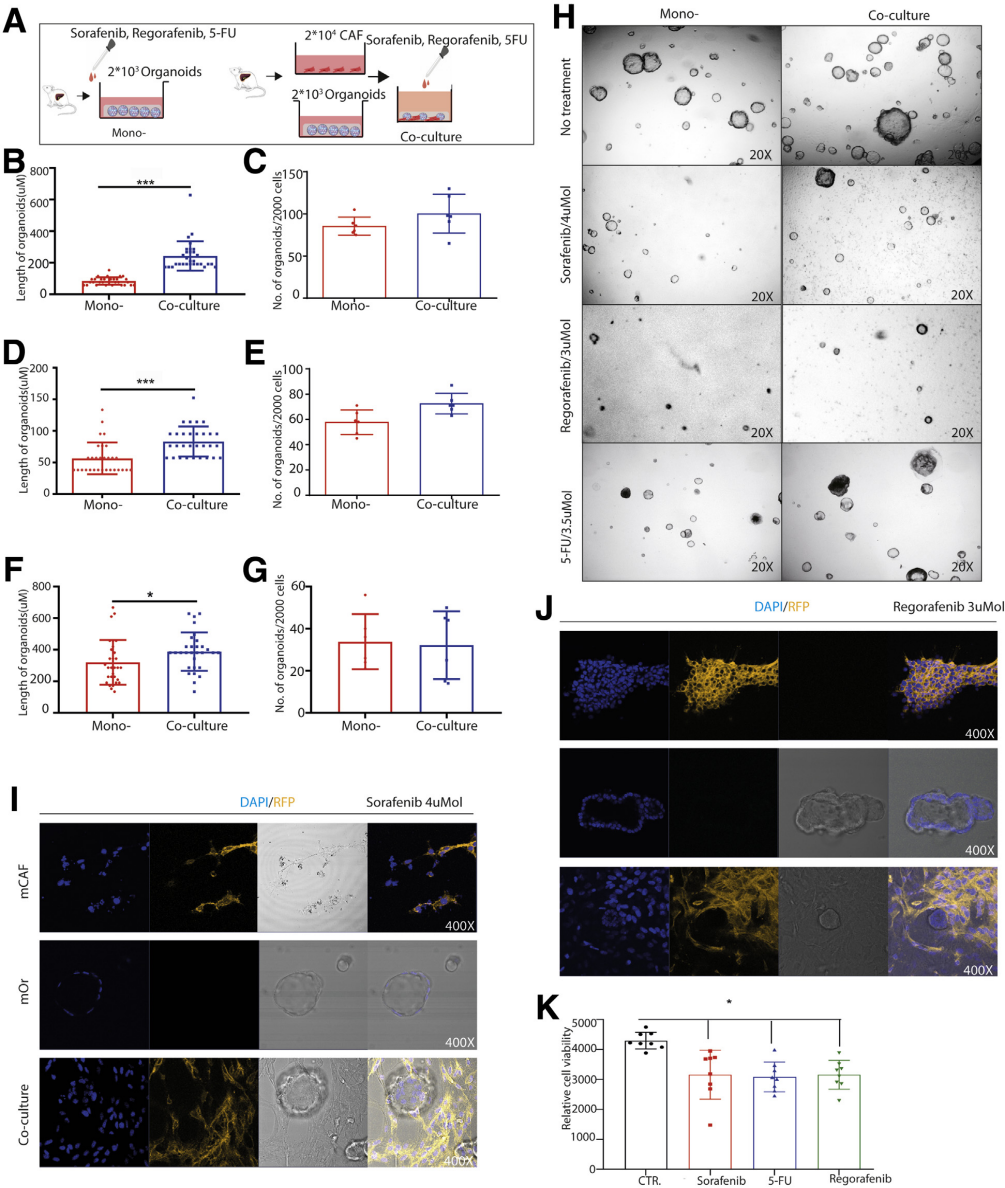
**Mouse organoids in the presence or absence of CAFs in response to anticancer drugs.** (*A*) An outline of the experimental strategy used to illustrate the drug administration on mouse tumor organoids with or without CAFs. (*B–G*) Mouse organoids in response to treatment of sorafenib (4 umol), regorafenib (3 umol), or 5-FU (3.5 umol) with or without CAFs (n = 6). (*H*) Representative image of treatment for mouse mono-culture and co-culture (magnification, 20×). (*I* and *J*) Representative confocal image of mouse CAFs, organoids, and co-cultures in response to treatment with sorafenib and regorafenib (magnification, 400×). (*K*) Mouse CAFs in response to anticancer drugs (sorafenib, 5 umol; regorafenib, 5 umol; 5-FU, 5 umol; n = 8). (*B*, *D*, and *F*) Five organoids for each well were measured randomly. (*B–G* and *K*) Data are presented as means ± SD, Mann–Whitney *U* tests. ∗*P* < .05, ∗∗∗*P* < .001. CTR, control; DAPI, 4′,6-diamidino-2-phenylindole; mCAF, mouse cancer associated fibroblast; mOR, mouse organoid; RFP, red fluorescent protein.

**Figure 12:**
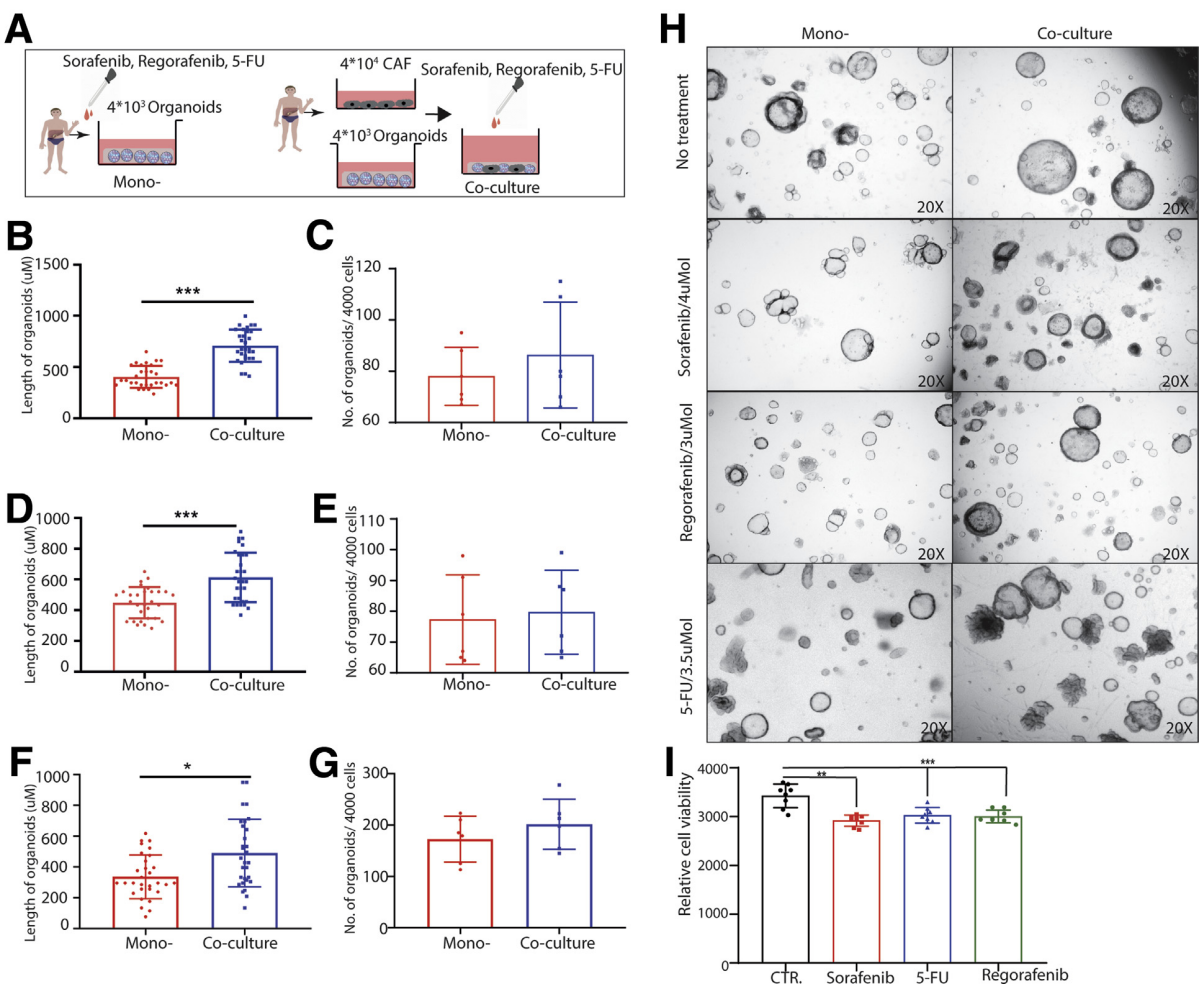
**Human organoids in the presence or absence of CAFs in response to anticancer drugs.** (*A*) An outline of the experimental strategy used to illustrate the drug treatment on human tumor organoids with or without CAFs. (*B–G*) Human organoids in response to treatment with sorafenib (4 umol), regorafenib (3 umol), or 5-FU (3.5 umol) with or without CAFs. (*H*) Representative image of human mono-culture and co-culture with or without treatment (magnification, 20×). (*I*) Human CAFs in response to anticancer drugs (sorafenib, 5 umol; regorafenib, 5 umol; 5-FU, 5 umol; n = 8). (*B*, *D*, and *F*) Five organoids for each well were measured randomly. (*B–G* and *I*) Data are presented as means ± SD, Mann–Whitney *U* tests. ∗*P* < .05, ∗∗*P* < .01, ∗∗∗*P* < .001. CTR, control.

We next examined the effects of CAFs on the response of tumor organoids to the anticancer drugs including sorafenib, regorafenib, and 5-fluorouracil (5-FU). Mouse liver tumor organoids were treated with sorafenib, regorafenib, or 5-FU in the presence or absence of CAFs (Figure 11*A*). Although the number of formed organoids was not significantly different, the diameters of organoids were significantly larger when co-culturing with CAFs compared with organoids alone (Figure 11*B–H*). Of note, most of the organoids that survived the treatment were surround by CAFs (Figure 11*I* and *J*). These results were confirmed further in human liver tumor organoids treated with sorafenib, regorafenib, or 5-FU in the presence or absence of human CAFs (Figure 12*A–H*). Of note, treatment with sorafenib, regorafenib, or 5-FU at 5 umol exerted moderate inhibition on cultured CAFs (Figures 11*K* and 12*I*).

**Figure 13:**
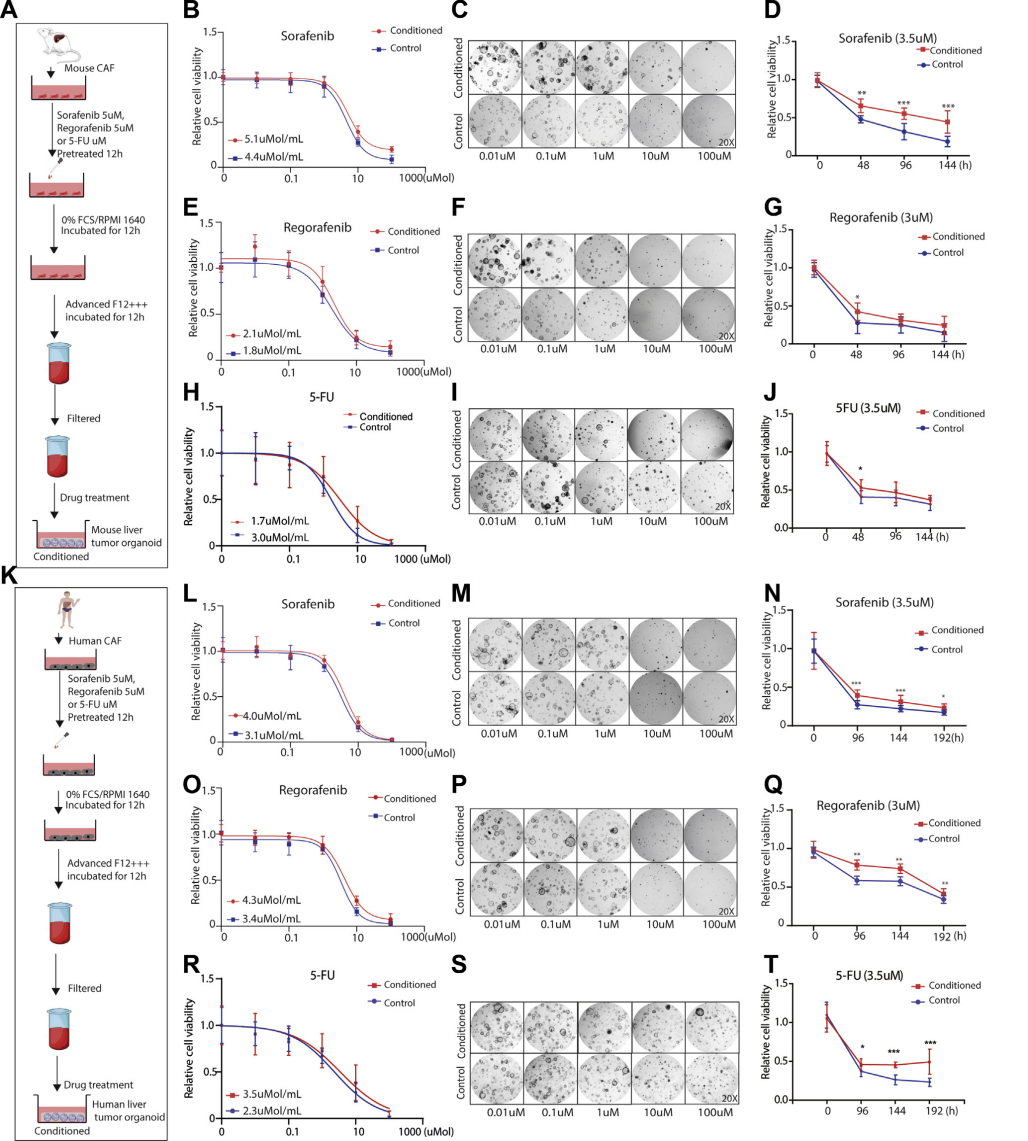
**Organoids in the presence or absence of CAF conditioned medium in response to the anticancer treatment.** (*A* and *K*) An outline of the experimental strategy used to illustrate drug treatment on tumor organoids with or without conditioned medium of pretreated CAFs. (*B*, *E*, *H*, *L*, *O*, and *R*) Organoids in the presence or absence of conditioned medium of pretreated CAFs were treated with a serial concentration of sorafenib, regorafenib, or 5-FU, and the half maximal inhibitory concentration was determined (n = 9; data are presented as means ± SD). (*C*, *F*, *I*, *M*, *P*, and *S*) Representative image of mouse or human tumor organoids in the presence or absence of conditioned medium of pretreated CAFs, treated with a serial concentration of sorafenib, regorafenib, or 5-FU for 10 days for mouse cells and 14 days for human cells (magnification, 20×). (*D*, *G*, *J*, *N*, *Q*, and *T*) Cell viability assays were performed and measured at the indicated times, using mouse or human tumor organoids incubated with the indicated anticancer drugs and parenthesized concentration in the presence or absence of conditioned medium of pretreated CAFs (n = 9). Graphs show means ± SD of data normalized to *t* = 0. Mann–Whitney *U* tests. ∗*P* < .05, ∗∗*P* < .01, ∗∗∗*P* < .001.

To investigate whether these effects are related to paracrine signaling, both mouse and human organoids were exposed to conditioned medium of CAFs and treated with sorafenib, regorafenib, or 5-FU (Figure 13*A* and *K*). Interestingly, organoids in the presence of CAF-conditioned medium are more resistant to treatment, as shown by higher half-maximal inhibitory concentrations (Figure 13*B*, *E*, *H*, *L*, *O*, and *R*) and the morphologic appearance (Figure 13*C*, *F*, *I*, *M*, *P*, and *S*). A dynamic response of treatment at different time points showed a similar pattern of resistance in the presence of CAF-conditioned medium (Figure 13*D*, *G*, *J*, *N*, *Q*, and *T*). Taken together, these findings show that CAFs protect tumor organoids from anticancer treatment.

## Discussion

CAFs as a vital component of the tumor microenvironment have been shown extensively to support cancer development and progression, and to promote treatment resistance.^24^^,^^25^ The clinical significance of CAFs in disease progression, therapeutic response, and patient outcome has been widely reported in various types of cancer.^26^, ^27^, ^28^ In this study, we found that enhanced expression of CAF markers in liver tumors were associated with poor patient outcome. Currently, a major challenge is how to dissect the interactions of cancer cells with CAFs in robust experimental models. We successfully established 3D co-culture systems of liver tumor organoids and CAFs of both mouse and human origins to study the interactions between these 2 cell types.

A prominent role of CAFs is thought to shape the stem cell niche to nurture CSCs, whereas the conventional 2-dimensional culture of immortalized cancer cell lines is far from satisfactory in recapitulating the properties of CSCs.^29^^,^^30^ The recent development of organoid technology, which grows embryonic or adult mammalian stem cell–derived 3D organotypic structures in vitro, has greatly facilitated stem cell research. This now has been extended to the culture of primary cancer cells that recapitulate the genomic and structural architecture of the tumor-of-origin, and especially the CSC compartment.^31^ Tumor organoids have been established successfully across a variety of cancer types, including liver cancer.^4^^,^^5^^,^^32^ The co-culture model of organoids with CAFs first was pioneered in pancreatic cancer because pancreatic cancer has the most extensive stromal reaction, accounting for up to 90% of the tumor volume.^33^^,^^34^ In this study, we established the co-culture of liver tumor organoids with CAFs. We first cultured organoids and CAFs from DEN-induced mouse liver tumors. We recently showed that these organoids can recapitulate the heterogeneity of patient liver cancer types to some extent.^5^ For patient liver cancer, organoids are much easier to be cultured from CCA compared with HCC,^4^ and therefore we used CCA organoids for establishing the model. Our model systems shall enable the detailed study of interactions between liver cancer cells, especially CSCs, with CAFs.

CAFs secrete a variety of cytokines, chemokines, and growth factors to create a tumor-permissive microenvironment.^35^ Many factors, such as chemokine ligand 5, C-X-C motif chemokine 12, transforming growth factor β, insulin-like growth factors (IGF), epidermal growth factors (EGF), fibroblast growth factors (FGF), interleukin (IL)6, IL8, IL10, and IL11, secreted by CAFs have an essential role in regulating cancer development.^26^^,^^29^^,^^36^, ^37^, ^38^, ^39^, ^40^, ^41^, ^42^ In addition to biochemical cross-talk, direct contact between CAFs and cancer cells also plays a critical role in tumor progression. By extracellular matrix remodeling, CAFs facilitate the migration of cancer cells.^20^ On the other hand, CAFs directly exert a pulling force on cancer cells through epithelial to mesenchymal transition by mediating N-cadherin and E-cadherin expression.^43^ These results are in accordance with our findings that CAFs confer a growth advantages of tumor organoids in co-culture with cell–cell contact and in a Transwell system via paracrine signaling. Furthermore, co-transplantation with CAFs promotes organoid-based tumor formation and growth in mice. In pancreatic tumor organoids, a wingless-related integration site (Wnt) nonproducing subtype requires Wnt ligands from CAFs.^33^ CAF-derived hepatocyte growth factor (HGF) has been reported to regulate liver tumor–initiating cells via activation of Fos-related antigen 1 in an extracellular signal-regulated kinase 1/extracellular signal-regulated kinase 12-dependent manner.^17^ In our model, the exact contribution of paracrine signaling and physical interaction, and the underlying molecular mechanisms, remain to be explored further.

Recruitment of fibroblasts to tumor stroma is regulated by multiple factors, which is highly context-dependent but remains not fully understood. It has been suggested that during tumor initiation, CAFs can be differentiated from the local fibroblast population of the epithelial stroma upon stimulation by transforming growth factor, whereas at later tumor progression stages CAFs are recruited mainly from distal locations.^44^, ^45^, ^46^ Thus, the origin of CAFs appears diverse and can be derived from different sources, such as tissue residual fibroblasts, bone marrow–derived cells, endothelial cells, pericytes, vascular smooth muscle cells, or even cancer cells that undergo epithelial to mesenchymal transition.^47^, ^48^, ^49^ However, most of the previous studies have suggested that CAFs are noncancer cells. In our study, we found that CAFs were negative for AFP and EpCAM, the markers that are expressed by liver tumor organoids. Furthermore, we did not observe tumor formation by transplanting a large number of CAFs into flanks of NOD scid γ mouse (NSG) immunodeficient mice. These results suggest that phenotypically and functionally our CAFs are not cancer cells. The different origins and different contexts may endow distinct phenotypes and functions of CAFs. This partially may explain our exploratory observation that in vitro cultured, transplanted, and in vivo spontaneously recruited CAFs express different patterns of CAF markers.

Development of drug-resistance is a relentless clinical challenge for cancer treatment.^50^ This re-enables tumor growth, cancer cell dissemination, and early onset of metastasis. Studies on the mechanisms of therapy resistance have focused primarily on the intrinsic properties of tumor cells. Emerging evidence has indicated the role of the organ/tumor-specific microenvironment for developing drug resistance. CAFs contribute to treatment resistance mainly through impaired drug delivery and biochemical signaling. Remodeled ECM by CAFs acts as a physical barrier to inhibit the uptake of anticancer drugs by increasing intratumoral interstitial fluid pressure and inducing vascular collapse.^51^^,^^52^ CAF-derived soluble factors including IL6, IL17A, IGF1, IGF2, and nitric oxide indirectly can mediate the development of cancer treatment resistance.^51^^,^^53^, ^54^, ^55^ Our study showed that co-culture with CAFs confer resistance of liver tumor organoids to the clinically used anticancer drugs including 5-FU, sorafenib, and regorafenib. This effect was recapitulated by adding conditioned medium from CAFs. However, whether this effect occurs in vivo and the involved molecular mechanisms remain to be studied further.

A recent study showed that the CAF population is implicated in immune dysregulation and is associated with immunotherapy outcome in melanoma patients.^56^ Interestingly, cultured CAFs from colon tumor, as well as lung cancer, have been reported to express immune checkpoint molecule programmed death 1 ligand 1/2, which strongly induce T-cell exhaustion.^57^^,^^58^ CAFs also indirectly may regulate the immune response through ECM remodeling by acting as a barrier that block the access of immune cells to cancer cells.^59^ A co-culture model with human pancreatic cancer organoids, matched stromal and immune cells recently was developed. Thus, we will further advance of our models by incorporating immune cells that will enable the study of tumor stroma and tumor immune interaction and the assessment of immunotherapeutics such as checkpoint inhibitors in the context of T-cell infiltration.^60^ Because the clinical benefits of immune-based therapies for HCC are evident, ongoing clinical trials soon will establish their role in the management of HCC patients.

In summary, we successfully have established 3D co-culture models of liver tumor organoids with CAFs of mouse or human origin. We have shown the robust effects of CAFs in liver cancer nurturing and treatment resistance. These model systems will be helpful for future research on the interactions of liver cancer cells with a stromal compartment and facilitate therapeutic development.

## Materials and Methods

### Mouse Liver Tumor Organoid Culture

Mouse liver tumor organoids were cultured from DEN-treated Leucine-rich repeat-containing G-protein coupled receptor 5-diphtheria toxin-green fluorescent protein mice with histologically verified liver tumors. Tumor tissue was minced and digested with a digestion solution: collagenase type XI (0.5 mg/mL, C9407; Sigma Aldrich, St. Louis, MO), dispase (0.2 mg/mL, 17105041; Sigma Aldrich), and 1% fetal bovine serum in Dulbecco’s modified Eagle medium (DMEM, 37°C, 30 minutes; Lonza, Basel, Halbkanton). The tissue debris was allowed to settle, and the dissociated cells were pelleted and washed in advanced DMEM/F12 (12634010; Invitrogen, Waltham, MA) and seeded in Matrigel (356231; BD Bioscience, Basel, Halbkanton). After the Matrigel became solid, expansion medium was added slowly. Mouse organoid expansion medium (OEM) was based on mouse organoid basic medium (OBM) (advanced DMEM/F12 supplemented with 1% penicillin/streptomycin [15140122; Life Technologies, Bleiswijk, South Holland], 1% GlutaMAX [BE-17-605E/U1; Westburg BV, Leusden, Zuid Holland], 10 mmol/L HEPES [be-17-737E; Westburg BV]), B27 (2% vol/vol, 17504-001; Life Technologies Europe BV), N2 (1%, vol/vol, 17502001; Life Technologies), N-acetylcysteine (1.25 μmol/L, A7250; Sigma-Aldrich), gastrin (10 nmol/L, G9145; Sigma Aldrich), EGF (50 ng/mL, AF-100-15; PeproTech, London), R-spondin 1 (10% vol/vol, conditioned medium produced by the R-Spondin1-expressing 293T cell line), FGF10 (100 ng/mL, 100-26; PeproTech), nicotinamide (10 mmol/L, N0636; Sigma-Aldrich), and HGF (50 ng/mL, 167100-39-0500; PeproTech). For the initial 3 days, the organoids were cultured with organoid initiation medium supplemented with noggin (10% vol/vol, conditioned medium produced by the Noggin-expression 293T cell line), Wnt3a (10% vol/vol, conditioned medium produced by the L-Wnt3a cell line), and Y-27632 (10.5 μmol/L, Y0503; Sigma-Aldrich).

For passaging, cold OBM was used to collect the organoids. Organoids were dissociated mechanically into small pieces by pipetting, and then seeded back into fresh Matrigel again. Passaging was performed at a ratio of 1:6∼1:10 per week according to the growth of the organoids. To create frozen stock, organoids were passaged and mixed with freeze medium (90% fetal bovine serum supplemented with 10% dimethyl sulfoxide) using standard procedures. Cultures were thawed using standard thawing procedures, washed once with OBM, and seeded in Matrigel (356231; Corning BV, Amsterdam, Zuid-Holland) with organoid initiation medium for the first passage.

### Isolation and Culture of Mouse CAFs

Mouse CAFs were isolated from DEN-induced Rosa26-membrane tomato mice with histologically verified liver tumors. CAFs were isolated by using an outgrowth isolation. Tissue from tumor edge was minced and digested with a digestion solution: collagenase type XI (0.5 mg/mL, C7657; Sigma Aldrich), dispase (0.2 mg/mL, 17105041; Sigmal-Aldrich), and 1% fetal bovine serum in DMEM (Lonza) for 30 minutes to 2 hours at 37°C in a water bath. Then the sample was filtered by using a filter tip and subsequently quenched in 10% fetal calf serum (FCS) RPMI 1640 medium. The pellet that contained tumor debris was plated in a T25 flask and fibroblast was allowed to grow and attach to the wall of the flask. To avoid cancer cell contamination, established cell culture was passaged at least 3 generations. The medium was changed every 2 days. CAFs were subcultured when reaching 80% confluence, banked, and used for experimental studies at passages 4–8. The fibroblasts were checked by using immunofluorescence staining of the fibroblast markers α-SMA (1:1000, ab124964; Abcam, Cambridge, Cambridgeshire), FAP (1:500, ab28244; Abcam), and negative staining for the HCC cell (AFP, 1:50, SAB3500533; Sigma-Aldrich), epithelial cell marker (EpCAM, 1:1000, ab71916; Abcam), endothelial marker (CD31, 1:50, ab28364; Abcam), and immune cell marker (CD45, 1:200, 13917; Cell Signalling, Danvers, MA) to exclude contamination of other cell types before being subjected to experiments.

### Human CCA Organoids and CAF Culture

OEM for culturing human CCA organoids was based on OBM, B27 (2% vol/vol), N2 (1% vol/vol; Invitrogen), N-acetylcysteine (1.25 μmol/L), gastrin (10 nmol/L), Rspo-1 conditioned medium (10% vol/vol), 10 mmol/L nicotinamide, recombinant human EGF (50 ng/mL), recombinant human FGF10 (100 ng/mL), recombinant human HGF (25 ng/mL), 10 μmol/L forskolin (1099; Bio-Techne, Minneapolis, MN), 5 μmol/L A8301 (2939/10; Bio-Techne), and 10 μmol/L Y27632. Upon attainment of dense tumor-derived organoids (2–3 weeks after isolation), they were passaged by mechanical dissociation into small fragments via trituration with a pipette, and transferred to fresh Matrigel in the previously defined OEM. Medium was refreshed every 2–3 days and organoids were passaged in a 1:2–1:10 split ratio according to the growth of the organoids. For isolation and culture of human CAFs from HCC and CCA tumors, the protocol was similar to isolation and culture of mouse CAFs.

The study was approved by the medical ethical committee of Erasmus Medical Center. In addition, the study protocol conforms to the ethical guidelines of the 1975 Declaration of Helsinki.

### Co-culture of Tumor Organoids and CAFs

Cold OBM was used to collect the organoids. Organoids were dissociated mechanically into small pieces by pipetting (20–30 times), and digested further into single cells by trypsin-EDTA (37°C, 2 minutes; Gibco). Fluorescence-activated cell sorting (BD FACS Aria II, San Jose, CA) was used to further isolate the single living cells. Propidium iodide staining was used to exclude dead cells. Forward scattered light–width with forward scattered light–area and then side scattered light–width with side scattered light–area gates were used to select the single cells. CAFs were collected when they were 80% confluent in the flask. After digesting into single cells, fluorescence-activated cell sorting was used to isolate the single living cells further. For co-cultures, different concentrations between CAFs and tumor organoid cells were sorted into 48-well or 96-well plates with OBM containing 1% Matrigel. Then the cells were centrifuged in 1000 rpm for 3 minutes and incubated on the plate overnight. The supernatant was removed on the second day and the plastic surface of the wells was coated with Matrigel to provide a biomatrix for 3D organoid growth. When the Matrigel became solid, mouse OEM or human OEM were added. After co-culturing organoid cells with CAFs of mouse origin for 7 days and those of human origin for 14 days, the diameters of organoids was measured using a scale tool from ZenLightEdition Software.

### Transwell Culture

For Transwell culture, 1000 CAF cells were seeded on top of the Transwell membrane (1-μm pore size, 662610; Greiner Bio-One, Alphen aan den Rijn, South Holland), and 500 single organoid cells growing in the lower compartment in 24-well plates for 10 days for mouse cells and 14 days for human cells.

### Alamar Blue Assay

CAFs or organoids were incubated with Alamar Blue (1:20 in DMEM, DAL1100; Invitrogen) for 2 hours (37°C), and then medium was collected for analysis of the metabolic activity of the cells. Absorbance was determined by using a fluorescence plate reader (CytoFluor Series 4000; Perseptive Biosystems, Framingham, MA) at an excitation of 530/25 nm and an emission of 590/35 nm. Matrigel with medium only was used as blank control.

### Cell Titer Assay

After culturing organoids for 10 days for mouse cells or 14 days for human cells in Transwell, a volume of CellTiter-Glo 3D reagent (G9681; Promega, Madison, WI) equal to the volume of cell culture medium was added in each well. The contents were mixed vigorously for 5 minutes to induce cell lysis. The plate was incubated at room temperature for an additional 25 minutes to stabilize the luminescent signal, and then the luminescence was recorded.

### Quantitative Real-Time Reverse-Transcription Polymerase Chain Reaction

Total RNA was isolated using the Macherey-Nagel NucleoSpin RNA II kit (Bioke, Leiden, South Holland) and quantified using a Nanodrop ND-1000 (Thermo Fisher, Wilmington, NC). Quantification was measured with a Nanodrop ND-1000. RNA then was converted to complementary DNA by using a complementary DNA Synthesis kit (Takara Bio, Saint-Germain-en-Laye). Real-time polymerase chain reactions (PCRs) were performed with SYBRGreen-based real-time PCR (Applied Biosystems) and amplified in a thermal cycler (GeneAmp PCR System 9700; Thermo Fisher). For cells collected from murine tissues, the *Gapdh* gene was used as a reference. All quantitative reverse-transcription PCR primers are listed in Table 1.

**Table 1:** All Quantitative Reverse-Transcription PCR Primers Applied in This Study

Name	Sequence
Human stem cell markers	
BMI1 F	GGTACTTCATTGATGCCACAACC
BMI1 R	CTGGTCTTGTGAACTTGGACATC
LGR5 F	CCTGCTTGACTTTGAGGAAGACC
LGR5 R	CCAGCCATCAAGCAGGTGTTCA
OCT4 R	CCTGAAGCAGAAGAGGATCACC
OCT4 F	AAAGCGGCAGATGGTCGTTTGG
CD133 R	CACTACCAAGGACAAGGCGTTC
CD133 F	CAACGCCTCTTTGGTCTCCTTG
CK7 R	TGTGGATGCTGCCTACATGAGC
CK7 F	AGCACCACAGATGTGTCGGAGA
HOPX R	ATTCCACCACGCTGTGCCTCAT
HOPX F	AGTCTGTGACGGATCTGCACTC
SOX2 R	GCTACAGCATGATGCAGGACCA
SOX2 F	TCTGCGAGCTGGTCATGGAGTT
LRIG1 R	GTGTCATCACCAACCACTTTGGC
LRIG1 F	GCAATCTGAGGGTTTGGGTGAC
TERT R	GCCGATTGTGAACATGGACTACG
TERT F	GCTCGTAGTTGAGCACGCTGAA
CD44 R	CCAGAAGGAACAGTGGTTTGGC
CD44 F	ACTGTCCTCTGGGCTTGGTGTT
NANOG R	CTCCAACATCCTGAACCTCAGC
NANOG F	CGTCACACCATTGCTATTCTTCG
Mouse stem cell markers	
BMI1 F	ACTACACGCTAATGGACATTGCC
BMI1 R	CTCTCCAGCATTCGTCAGTCCA
LGR5 F	AGAGCCTGATACCATCTGCAAAC
LGR5 R	TGAAGGTCGTCCACACTGTTGC
OCT4 R	CAGCAGATCACTCACATCGCCA
OCT4 F	GCCTCATACTCTTCTCGTTGGG
CD133 R	CTGCGATAGCATCAGACCAAGC
CD133 F	CTTTTGACGAGGCTCTCCAGATC
CK7 R	CGGAGATGAACCGCTCTATCCA
CK7 F	CATGAGCATCCTTGATTGCCAGC
SOX2 R	AACGGCAGCTACAGCATGATGC
SOX2 F	CGAGCTGGTCATGGAGTTGTAC
LRIG1 R	TTCAGCCAACGCTACCCTCACA
LRIG1 F	TAAGCCAGGTGATGCGTGGTGT
TERT R	GAAAGTAGAGGATTGCCACTGGC
TERT F	CGTATGTGTCCATCAGCCAGAAC
CD44 R	CGGAACCACAGCCTCCTTTCAA
CD44 F	TGCCATCCGTTCTGAAACCACG
NANOG R	GAACGCCTCATCAATGCCTGCA
NANOG F	GAATCAGGGCTGCCTTGAAGAG
MEX3A R	AGAGCCTCACGCAACAAGTCTG
MEX3A F	CTGGATGCGTTTGATGGTCGCT
MUC5AC R	CCACTTTCTCCTTCTCCACACC
MUC5AC F	GGTTGTCGATGCAGCCTTGCTT
SOX17 R	GCCGATGAACGCCTTTATGGTG
SOX17 F	TCTCTGCCAAGGTCAACGCCTT
SOX9 R	CACACGTCAAGCGACCCATGAA
SOX9 F	TCTTCTCGCTCTCGTTCAGCAG
MUC1 R	AGTGCCTCTGACGTGAAGTCAC
MUC1 F	GGGAGGGAACTGCATCTCATTC
OLFM4 R	GCCTCCAAAAGTGACCTTGTGC
OLFM4 F	TGCGTGTGCTGGTGGAAAAGAG
HOPX F	GGGTGCTTGTTGACCTTGTT
HOPX R	TCTCCATCCTTAGTCAGACGC
MGAPDH F	CATCACTGCCACCCAGAAGACTG
MGAPDH R	ATGCCAGTGAGCTTCCCGTTCAG
HGAPDH F	GTCTCCTCTGACTTCAACAGCG
HGAPDH R	ACCACCCTGTTGCTGTAGCCAA
Human CAF marker	
FAP R	GGAAGTGCCTGTTCCAGCAATG
FAP F	TGTCTGCCAGTCTTCCCTGAAG
α-SMA R	CTATGCCTCTGGACGCACAACT
α-SMA F	CAGATCCAGACGCATGATGGCA
VIMENTIN R	AGGCAAAGCAGGAGTCCACTGA
VIMENTIN F	ATCTGGCGTTCCAGGGACTCAT
FSP1 R	CAGAACTAAAGGAGCTGCTGACC
FSP1 F	CTTGGAAGTCCACCTCGTTGTC
PDGFRA R	GACTTTCGCCAAAGTGGAGGAG
PDGFRA F	AGCCACCGTGAGTTCAGAACGC
PDGFRB R	TGCAGACATCGAGTCCTCCAAC
PDGFRB F	GCTTAGCACTGGAGACTCGTTG
CD29 R	GGATTCTCCAGAAGGTGGTTTCG
CD29 F	TGCCACCAAGTTTCCCATCTCC
CAV1 R	CCAAGGAGATCGACCTGGTCAA
CAV1 F	GCCGTCAAAACTGTGTGTCCCT
DESMIN R	CTGAGCAAAGGGGTTCTGAG
DESMIN F	ACTTCATGCTGCTGCTGTGT
GREMLIN1 R	TCATCAACCGCTTCTGTTACGGC
GREMLIN1 F	CAGAAGGAGCAGGACTGAAAGG
COL1A1 R	GATTCCCTGGACCTAAAGGTGC
COL1A1 F	AGCCTCTCCATCTTTGCCAGCA
PERIOSTIN R	TGCCCAGCAGTTTTGCCCAT
PERIOSTIN F	CGTTGCTCTCCAAACCTCTA
Mouse CAF marker	
GREMLIN1 R	AGGTGCTTGAGTCCAGCCAAGA
GREMLIN1 F	TCCTCGTGGATGGTCTGCTTCA
COL1A1 R	CCTCAGGGTATTGCTGGACAAC
COL1A1 F	CAGAAGGACCTTGTTTGCCAGG
PERIOSTIN R	CAGCAAACCACTTTCACCGACC
PERIOSTIN F	AGAAGGCGTTGGTCCATGCTCA
VIM F	CGGAAAGTGGAATCCTTGCAGG
VIM R	AGCAGTGAGGTCAGGCTTGGAA
FSP1 F	AGCTCAAGGAGCTACTGACCAG
FSP1 R	GCTGTCCAAGTTGCTCATCACC
CD29 F	CTCCAGAAGGTGGCTTTGATGC
CD29 R	GTGAAACCCAGCATCCGTGGAA
CAV1 F	CACACCAAGGAGATTGACCTGG
CAV1 R	CCTTCCAGATGCCGTCGAAACT
Desmin F	GCGGCTAAGAACATCTCTGAGG
Desmin R	ATCTCGCAGGTGTAGGACTGGA
FAP F	CACCTGATCGGCAATTTGTG
FAP R	CCCATTCTGAAGGTCGTAGATGT
α-SMA F	CCAGAGCAAGAGAGGGATCCT
α-SMA R	TGTCGTCCCAGTTGGTGATG
PDGFRA F	GCAGTTGCCTTACGACTCCAGA
PDGFRA R	GGTTTGAGCATCTTCACAGCCAC
PDGFRB F	GTGGTCCTTACCGTCATCTCTC
PDGFRB R	GTGGAGTCGTAAGGCAACTGCA

BMI, body mass index; CAV1, caveonin 1; CD, cluster of differentiation; CK7, cytokeratin 7; COL1A1, collagen type I a 1; F, forward; FSP1, fibroblast-specific protein 1; HGAPDH, human glyceraldehyde 3-phosphate dehydrogenase; HOPX, Homeodomain-only protein; LGR, Leucine-rich repeat-containing G-protein coupled receptor; LRIG1, Leucine-rich repeats and immunoglobulin-like domains protein 1; MEX3A, Mex-3 RNA Binding Family Member A; MGAPDH, mouse Glyceraldehyde 3-phosphate dehydrogenase; MUC, mucin; NANOG, Tir Na Nog; OCT4, octamer-binding transcription factor 4; OLFM4, Olfactomedin 4; PDGFRB, platelet-derived growth factor receptor b; R, reverse; SOX, SRY-Box Transcription Factor; TERT, Telomerase reverse transcriptase; VIM, vimentin.

### Organoid-Based Tumor Formation Assay in NSG Mice

Five- to 6-week-old NSG immunodeficient mice were used for the in vivo tumorigenesis assay. Mouse or human organoids (2.5 × 10^5^) together with or without 2.5 × 10^5^ CAFs in 100 uL Matrigel subcutaneously were inoculated into the flanks of the mice. A total of 2.5 million CAFs alone were injected as control. Tumor formation and tumor weight were examined and determined after 1–2 months. Mice were housed in a room maintained on a 12-hour light/dark cycle (light on at 6 am) with food and water provided ad libitum. All animal experiments were approved by the Committee on the Ethics of Animal Experiments of the Erasmus Medical Center.

### Flow Cytometry Assay and Cell Sorting

For FACS analysis, single cells derived from liver and organoids were suspended in DMEM plus 2% fetal bovine serum. Cell suspensions were analyzed using a BD FACSCalibur (BD Biosciences, San Jose, CA) or BD FACSAria II. For FACS, a BD FACSAria II cell sorter was used to isolate the target cell population. Single-cell suspensions of tumor cells were labeled with R-phycoerythrin (PE) anti-human CD140a antibody (PDGFRA, 5 μL per million cells in 100 μL volume, 323506; BioLegend, San Diego, CA), Pacific Blue anti-human CD31 antibody (2 μL per million cells in 100 μL volume, 102422; BioLegend), fluorescein isothiocyanate anti-human CD326 antibody (EpCAM, 5 μL per million cells in 100 μL volume, 324204; BioLegend), Alexa Fluor 700 anti-human CD45 antibody (1 μL per million cells in 100 μL volume, 135906; BioLegend), and PE anti-mouse CD140a antibody (PDGFRA, 5 μL per million cells in 100 μL volume, 135906; BioLegend). For cell sorting, PDGFRA+ for CAFs were collected and processed for RNA extraction and quantitative reverse-transcription PCR.

### Immunofluorescence

CAFs were fixed in 4% paraformaldehyde for 1 hour and permeabilized by incubation in phosphate-buffered saline (PBS) with a concentration of 0.2% Triton X-100 (Sigma-Aldrich) at room temperature for 20 minutes. Samples were blocked for 1 hour at room temperature in blocking buffer: 5% bovine serum albumin PBS 0.05% Tween 20 (P9416; Sigma-Aldrich). Then cells were incubated with primary antibody anti–α-SMA (1:1000, ab124964; Abcam), anti-FAP (1:500, ab28244; Abcam), anti-EpCAM (1:1000, Ab71916; Abcam), anti-AFP (1:50, SAB3500533; Sigma-Aldrich), anti-CD31 (1:50, ab28364; Abcam), and anti-CD45 (1:200, 13917s; Cell Signaling) in blocking solution in a wet chamber overnight at 4°C. After 3 washes of 15 minutes in PBS, the samples were mounted and analyzed using a Zeiss LSM510meta confocal microscope (Oberkochen, Baden-Wurttemberg).

### Tissue Histology, Immunohistology, and Immunofluorescence

For histologic analysis, tumors were dissected into 10% neutral buffered formalin, embedded in paraffin blocks, and serial sections were taken. Paraffin-embedded tissue sections were rehydrated before antigen retrieval using pH 6 sodium citrate buffer. After blocking endogenous peroxidase (DAKO peroxidase block, S202386-2; Agilent, Santa Clara, CA), sections were incubated with primary antibodies anti–α-SMA (1:1000, ab124964; Abcam) and anti-EpCAM (1:1000, Ab71916; Abcam) overnight. Then sections were incubated with a second antibody for 1 hour at room temperature. The slides were placed in 3,3′-diaminobenzidine tetra hydrochloride substrate (ab64238; Abcam) and incubated until the desired color was achieved (30 seconds to 3 minutes). Consequently, the slides were counterstained with hematoxylin. Images were acquired with a Zeiss Axioskop 20 microscope.

For immunofluorescence, samples were dehydrated further with 30% sucrose (S0389, 4°C, overnight; Sigma-Aldrich), stored at -80°C, and then sectioned at 8 μm for further analysis. Images were acquired with a Zeiss LSM510meta confocal microscope.

### Drug Treatment

Organoids and CAFs were digested by using trypsin-EDTA in single cells. By using FACS, 2000 mouse organoids or 4000 human organoid cells with or without CAFs in a 1:10 ratio were seeded and treated with sorafenib (4 umol/mL, SC-357801A; Bio-connect BV, Huissen, South Holland), regorafenib (3 umol/mL, S1178; Bio-connect BV), and 5-FU (3.5 umol/mL, F-6627; Sigma Aldrich) for 7 days. The number of formed organoids was counted and their diameters were measured.

To investigate the paracrine effect of CAFs on tumor organoids, conditional medium of CAFs (approximately 70% confluent) was collected. To recapitulate the effects of anticancer drugs on CAFs in co-culture models, CAFs were primed by pretreating with 5 umol/L sorafenib, 5 umol/L regorafenib, or 5 umol/L 5-FU in 10% FCS RPMI 1640 medium for 12 hours. Then the supernatant was removed and the cells were washed with PBS 3 times. CAFs then were cultured in 0% FCS RPMI 1640 medium for another 12 hours. After removing medium and washing 3 times with PBS, 10 mL OBM was added and conditioned for 12 hours. The supernatants then were collected and filtered with a 40-um filter.

For half-maximal inhibitory concentration analysis, 5000 mouse organoids or 10,000 human organoid cells were cultured with conditioned or unconditioned medium with a series of concentrations of sorafenib, regorafenib, or 5-FU for 10 days for mouse cells and 14 days for human cells. Cell viability was measured by the Alamar blue assay. To study the dynamic response of treatment at different time points, 5000 mouse or human organoid cells were cultured with conditioned or unconditioned medium with sorafenib (3.5 umol), regorafenib (3 umol), and 5-FU (3.5 umol) for 0 to 192 hours. The Alamar blue assay was used to determine cell viability.

### Online Database

We used a database of Gene expression profiling interactive analysis (GEPIA, Peking University, Beijing) gene expression profiling interactive analysis (http://gepia.cancer-pku.cn)^61^ to evaluate the association of patient overall survival with the expression of target genes in tumor and normal liver tissue as well as at different stages of tumors.

### Statistics Analysis

Prism software (GraphPad software 8.0; San Diego, CA) was used for all statistical analyses. Data were presented as means ± SD. For comparing gene expression in tumor tissue and surrounding tissue from an online database, 1-way analysis of variance was used to compare the difference between groups. For the overall survival rate between high and low expression of genes, log-rank or Kaplan–Meier was used to compare the differences between groups. For statistical significance of the differences between groups, we used the Mann–Whitney *U* test. Differences were considered significant at a *P* value less than .05.

All authors had access to the study data and reviewed and approved the final manuscript.
